# Genetic polymorphisms and early responses predict refractory hyperthyroidism during antithyroid drug therapy

**DOI:** 10.1016/j.isci.2026.117780

**Published:** 2026-10-08

**Authors:** Xinpan Wang, Mengyi Gong, Jiayan Wang, Qiuyi Wang, Kuanfeng Xu, Qi Fu, Yun Cai, Zhixiao Wang, Xiaoyun Liu, Xinyu Xu, Tao Yang, Xuqin Zheng

**Affiliations:** 1 Department of Endocrinology, The First Affiliated Hospital with Nanjing Medical University, Nanjing, China

**Keywords:** Graves’ disease, antithyroid drug, refractory, dose correction, early therapeutic response, genetic polymorphisms, treatment duration

## Abstract

Antithyroid drugs (ATDs) are the first-line treatment for Graves’ disease (GD), but variable outcomes require early risk stratification. We analyzed 578 adults, including 240 in a randomized trial of dose titration versus modified partial block-and-replace therapy and the remainder in a temporal observational cohort. Baseline characteristics, 3-month treatment indicators, and 13 GD-susceptibility single nucleotide polymorphisms (SNPs) were evaluated. An ATD-levothyroxine dose-correction method quantified thyroid function and autoantibody changes, informing clinical and SNP risk score-integrated models. The integrated model achieved areas under the curve (AUCs) of 0.841 in development and 0.805 in temporal validation, outperforming the clinical model (*p* < 0.001) with good calibration and net benefit. Intermediate-risk patients derived the greatest incremental benefit. Baseline free thyroxine (FT4) was the strongest predictor, and the SNP risk score ranked fourth. No inter-regimen efficacy or safety differences were observed. Restricted cubic spline analysis suggested that ATD therapy beyond 3 years may reduce recurrence risk, whereas modified partial block-and-replace showed no advantage over dose titration.

## Highlights

• An integrated model predicts refractory Graves’ disease with useful discrimination
• Dose-corrected early responses improve risk assessment during ATD therapy
• A candidate SNP risk score adds genetic information for East Asian patients
• ATD therapy beyond 3 years may be linked to lower recurrence risk

## Graphical abstract

**Figure:**
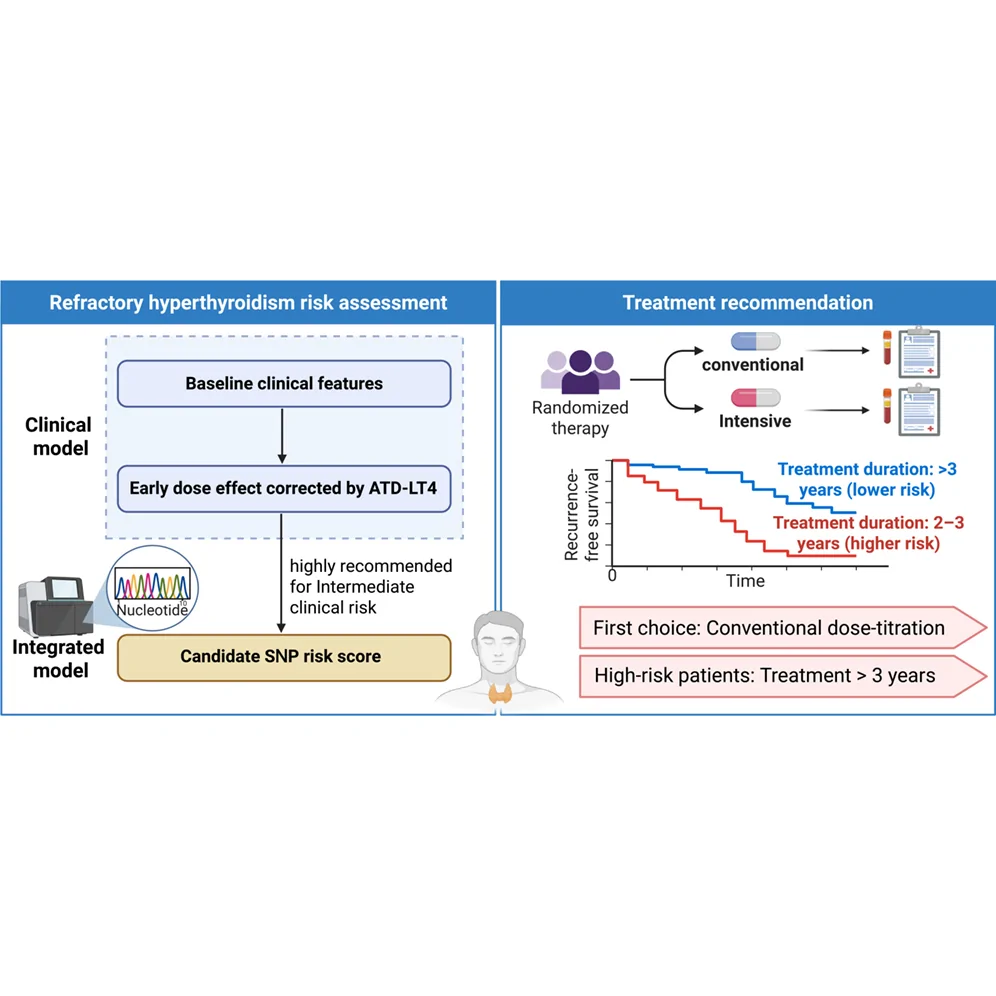

## Teaser

Health sciences

## Introduction

Graves’ disease (GD), also known as diffuse toxic goiter, is the most common cause of hyperthyroidism.^1^ Clinically, antithyroid drugs (ATDs), represented by methimazole (MMI), are generally administered as the first-line therapy.^2^^,^^3^ However, this therapeutic regimen is characterized by a high recurrence rate after drug withdrawal; approximately half of the patients require transition to radical therapies such as radioactive iodine (RAI) treatment or thyroidectomy due to recurrent episodes, resulting in a substantial increase in disease burden.^3^ Our research group previously proposed and established a precise, comprehensive definition for ATD-refractory GD: patients who either experience relapse after ATD withdrawal or fail to achieve thyroid-stimulating hormone (TSH) receptor antibody (TRAb) negativity and meet standard treatment cessation criteria despite >2 years of regular ATD therapy.^4^ Early identification of such high-risk patients is essential for optimizing clinical management.

ATD therapy comprises three distinct phases: the initial phase, the dose-reduction phase, and the maintenance phase. Two principal treatment strategies are commonly employed: block replacement and dose titration. Most previous studies investigating the block-replacement strategy have used a fixed-dose regimen throughout therapy.^5^^,^^6^ However, the conventional dose-titration approach often leads to thyroid function fluctuations due to imprecise dose adjustments, while a full-course, high-dose block-replacement strategy may disrupt normal physiological feedback mechanisms by excessively suppressing thyroid function.^5^^,^^7^^,^^8^ We previously conducted a prospective randomized controlled trial (RCT) comparing a modified partial block-replacement regimen (intensive therapy) with conventional dose-titration therapy (unpublished data). The primary outcome of the original RCT was limited to post-withdrawal recurrence only and did not capture patients with prolonged TRAb positivity (>2 years) who remained ineligible for treatment cessation. The present study represents a secondary analysis of this RCT, with a revised, clinically relevant primary endpoint—the overall rate of refractory GD—incorporating both post-withdrawal recurrence and failure to achieve treatment cessation despite prolonged ATD therapy.

In addition to comparing treatment strategies, this secondary analysis further explored predictive factors associated with refractory GD to facilitate early risk stratification. Preliminary studies conducted by our research group demonstrated that dynamic changes in thyroid hormones and thyroid autoantibodies during the early treatment phase (within 3 months), when integrated with baseline clinical characteristics, have greater prognostic value.^4^ However, these factors alone cannot fully explain the underlying causes of poor responses to standardized ATD therapy in certain patients. Beyond clinical phenotypes, genetic factors may serve as key regulators of heterogeneity in treatment responses.^9^

Genetic research has clearly established that GD has a significant hereditary component.^10^ Genome-wide association studies (GWASs) have identified multiple GD-susceptible single nucleotide polymorphism (SNP) loci, including *HLA-DPB1*, *CD40*, *CTLA4*, and *TSHR*.^11^^,^^12^ These genetic polymorphisms not only influence disease susceptibility but may also modulate ATD treatment responses and prognosis by regulating immune response pathways.^13^ Most existing genetic prognostic models for GD, including the Graves’ Recurrent Events After Therapy Plus (GREAT+) score, are based on European and American Caucasian populations, and their findings may not be fully applicable to Asian populations.^14^^,^^15^^,^^16^ Furthermore, there remains a lack of systematic evaluation of polygenic risk factors combined with early-treatment response indicators for prognostic prediction. Accordingly, this secondary analysis aimed to integrate baseline clinical characteristics, early treatment response dynamics, and East Asian-adapted genetic polymorphisms to develop and validate a clinically translatable integrated predictive model for refractory GD and, thus, support individualized risk stratification and optimized intervention strategies.

## Results

### Participants

The overall study enrolled a total of 578 treatment-naive adult GD patients. The model development cohort was derived from a previously conducted RCT of ATD regimens (2018–2020). A total of 240 treatment-naive patients were initially enrolled. After excluding 24 patients who did not adhere to the study protocol, 216 patients were included in the model development cohort. The reasons for exclusion were as follows: 3 cases of thyroidectomy due to thyroid carcinoma, 2 cases of spontaneous hypothyroidism, 5 cases of transition to RAI therapy, 1 case of switching to propylthiouracil due to MMI allergy, 5 cases of switching between therapy groups, 1 case of poor follow-up compliance, 5 cases of irregular medication intake, 1 case of voluntary withdrawal, and 1 case of loss to follow-up. The refractory GD rate was 42.6% (92/216). Among these refractory cases, 67.4% (62/92) were due to recurrence after drug withdrawal, whereas 32.6% (30/92) involved patients with persistently positive TRAb who did not meet withdrawal criteria despite receiving continuous therapy for more than 2 years.

A total of 338 treatment-naive patients were initially enrolled in the single-center temporal validation cohort (non-RCT, 2021–2023), and 130 patients were excluded. The exclusion criteria were as follows: 4 cases of thyroidectomy due to thyroid carcinoma, 6 cases of transition to RAI therapy, 13 cases of using or switching to non-protocol medications, 9 cases of pregnancy during the treatment period, 13 cases of failure to complete the 2-year therapy and meet withdrawal criteria, 29 cases of follow-up duration less than 1 year after drug withdrawal, 31 cases of irregular follow-up or treatment, and 25 cases of loss to follow-up. Ultimately, 208 patients were included in the validation cohort. The flow diagrams of patient recruitment for both cohorts are presented in Figure 1.

**Figure 1:**
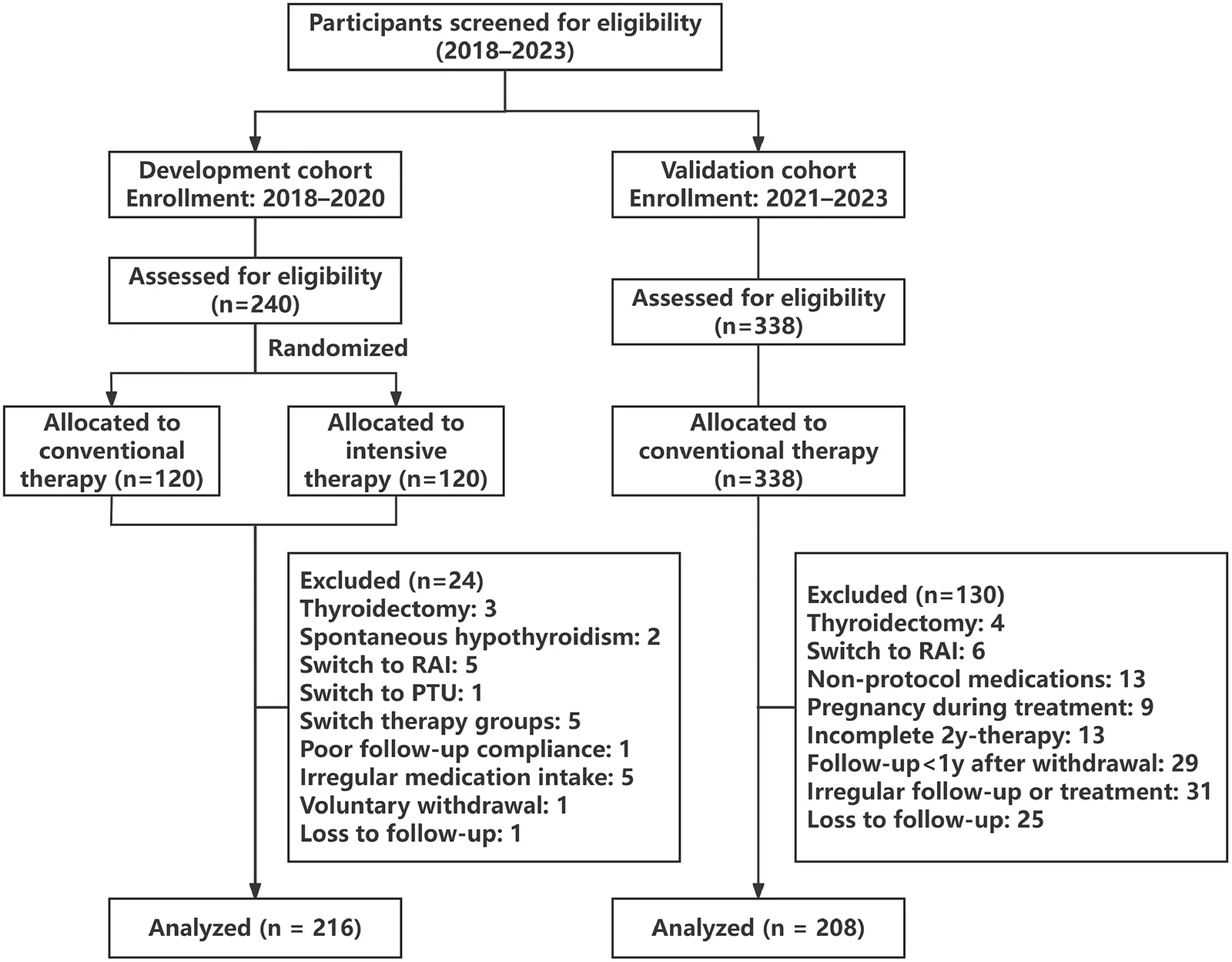
Flow diagram of patient enrollment in the development and validation cohorts PTU, propylthiouracil; RAI, radioactive iodine; y, year.

Analysis of baseline clinical characteristics in the development cohort revealed that, compared with non-refractory patients, refractory patients had a significantly lower mean age (*p* = 0.001), larger thyroid volume (*p* = 0.027), and higher baseline levels of free triiodothyronine (FT3, *p* = 0.023), free thyroxine (FT4, *p* = 0.010), and thyroid peroxidase antibody (TPOAb, *p* = 0.05). No significant between-group differences were observed in other baseline indicators, including sex and smoking status (Table 1). In addition, baseline characteristics were well balanced between the different ATD treatment subgroups (Tables S1 and S2).

**Table 1:** Baseline clinical characteristics of patients in the refractory and non-refractory groups (development cohort)

Baseline clinical characteristics	Refractory (*N* = 92)	Non-refractory (*N* = 124)	*p* value
Age, y	34 ± 10	39 ± 11	0.001
Female, *n* (%)	72 (78.3%)	97 (78.2%)	0.995
Intensive therapy, *n* (%)^a^	48 (52.2%)	61 (49.2%)	0.665
Current smoking, *n* (%)	8 (8.7%)	10 (8.1%)	0.868
GD family history, *n* (%)	11 (12.0%)	12 (9.7%)	0.591
GO, *n* (%)	22 (23.9%)	21 (16.9%)	0.204
Baseline thyroid volume (cm^3^)	18.4 (13.3, 25.4)	16.4 (12.5, 22.0)	0.027
Baseline FT3 (pmol/L)	27.4 (18.1, 39.3)	22.2 (15.6, 32.2)	0.023
Baseline FT4 (pmol/L)	76.1 (46.7, 100.0)	58.3 (43.6, 80.7)	0.010
Baseline TSH (mIU/L)	0.005 (0.005, 0.005)	0.005 (0.005, 0.005)	0.553
Baseline TPOAb (IU/mL)	166.4 (27.1, 315.5)	87.2 (18.1, 221.8)	0.050
Baseline TgAb (IU/mL)	174.0 (26.8, 506.3)	152.2 (23.5, 448.1)	0.805
Baseline TRAb (IU/L)	9.7 (4.1, 23.4)	8.2 (4.0, 17.2)	0.148

Continuous data are presented as mean ± SD or median (interquartile range [IQR]) as appropriate. Categorical data are presented as *n* (%). ^a^Intention-to-treat (ITT) group as assigned at baseline. FT3, free triiodothyronine; FT4, free thyroxine; GD, Graves’ disease; GO, Graves’ orbitopathy; TgAb, thyroglobulin antibody; TPOAb, thyroid peroxidase antibody; TRAb, thyroid-stimulating hormone receptor antibody; TSH, thyroid stimulating hormone.

Briefly, comparative efficacy and safety outcomes between the two ATD regimens in the original RCT were generally comparable, with no significant between-group differences observed in the rates of refractory hyperthyroidism, post-withdrawal relapse, persistent medication requirement, or adverse events. Detailed analyses of the original RCT are provided in supplemental information, with corresponding results summarized in Tables S3 and S5.

SNP genotyping results from the development cohort indicated that the genotype distribution of rs1883832 at the *CD40* locus differed significantly between the refractory and non-refractory groups (*p* < 0.001) (Table S6). In the development cohort, baseline TSH levels were below the lower limit of detection (0.005 mIU/L) in 94.4% (204/216) of patients; therefore, TSH was excluded from interaction and multicollinearity analyses due to extreme distribution skewness and a high proportion of values below the detection limit. No significant interactions were identified among the remaining baseline indicators (*p* > 0.05), and no evidence of multicollinearity was detected (variance inflation factor [VIF] < 10). Similarly, analysis of baseline clinical and genetic SNP characteristics in the validation cohort revealed no significant interactions among indicators and no multicollinearity among the variables (VIF < 10). Baseline clinical characteristics and SNP distributions in the validation cohort are presented in Tables S7 and S8.

### Baseline and early-therapy clinical risk factors

Univariate analysis revealed that a younger age at GD diagnosis (≤30 years), larger baseline thyroid volume (>28.0 cm^3^), higher baseline FT3 (>25.0 pmol/L), higher baseline FT4 (>72.1 pmol/L), higher baseline TPOAb (>170.9 IU/mL), and higher baseline TRAb (>20.6 IU/L) were all significantly associated with refractory GD (*p* < 0.05). Other baseline parameters, including sex and therapy group, showed no significant associations (*p* > 0.05) (Table S9).

At 3 months of therapy, significant differences were observed between the refractory and non-refractory groups in thyroid function, autoantibody levels, and cumulative drug exposure (*p* < 0.05) (Table S10). Univariate analysis indicated that refractory patients exhibited poorer early treatment responsiveness; the absolute dose-effect values of TSH and TRAb, as well as the relative dose-effect values of FT3, FT4, TSH, TPOAb, and TRAb, were significantly lower in the refractory group than in the non-refractory group (*p* < 0.05) (Table S11). Considering the impact of medications on alterations in thyroid function, relative dose-effect indicators at 3 months of therapy were used in subsequent analyses. The standardized correction method for ATD-LT4 is detailed in STAR Methods.

To identify the final set of clinical predictors, baseline and early-treatment variables were subsequently screened jointly. Thirteen variables with *p* < 0.1 in univariable logistic regression were entered into LASSO regression with 10-fold cross-validation (λ_1__SE_= 0.0655). Eight key clinical variables were retained: age, baseline thyroid volume, baseline FT4, baseline TPOAb, baseline TRAb, and 3-month relative dose-effect values of FT3, TPOAb, and TRAb (Figures 2A and 2B).

**Figure 2:**
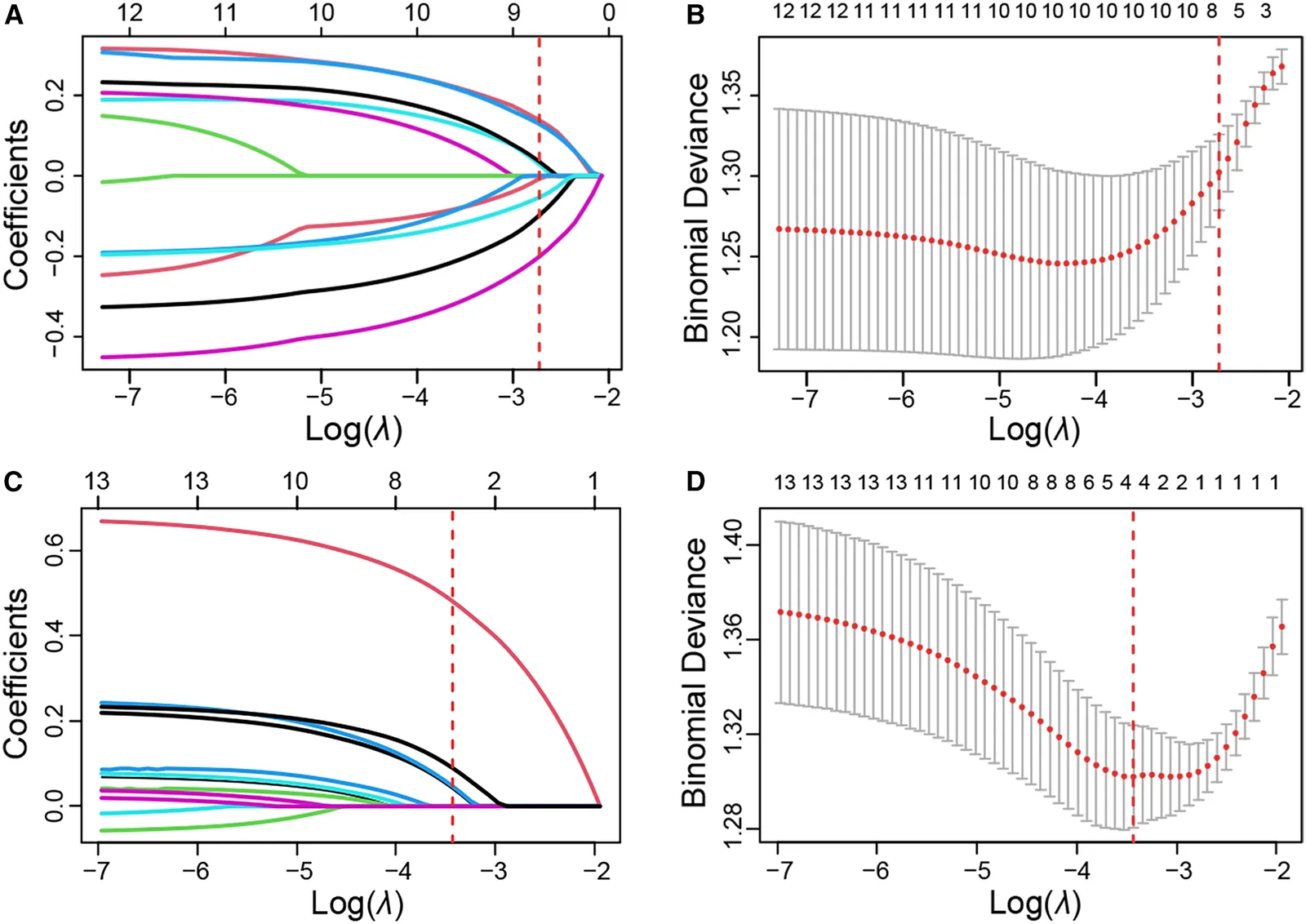
LASSO regression for variable selection of clinical and genetic predictors, analyzed in the development cohort (*N* = 216) (A) Coefficient path plot for clinical predictors. (B) Cross-validation plot for clinical predictors (*λ*_1SE_ = 0.0655). (C) Coefficient path plot for single-nucleotide polymorphisms (SNPs) associated with refractory Graves’ disease (GD). (D) Cross-validation plot for SNPs associated with refractory GD (*λ*_min_ = 0.032). LASSO, least absolute shrinkage and selection operator.

### Genetic risk factors and candidate SNP risk score

To identify genetic markers significantly associated with refractory hyperthyroidism, LASSO regression analysis with 10-fold cross-validation (*λ*_min_ = 0.032) was performed on 13 previously reported GD-susceptible SNPs.^17^^,^^18^^,^^19^^,^^20^ Four candidate gene loci were ultimately identified: *CD40*, *HLA-DPB1*, *LPP*, and *VANGL2* (Table 2; Figures 2C and 2D).

**Table 2:** SNP composition of the candidate SNP risk score

SNP	Risk allele	AF refractory	AF non-refractory	*β* (log(OR))	*p* value
*CD40* rs1883832	C	0.78	0.60	1.058	<0.001
*HLA-DPB1* rs2281388	A	0.53	0.48	0.388	0.124
*LPP* rs6780858	A	0.68	0.64	0.368	0.113
*VANGL2* rs117201373	G	0.19	0.13	0.515	0.079

AF, allele frequency; OR, odds ratio; SNP, single nucleotide polymorphism.

The candidate SNP risk score was constructed using the following formula (genotypes were assigned values of 0, 1, or 2 based on the number of risk alleles present):

SNPriskscore=1.058×CD40+0.388×HLA−DPB1+0.368×LPP+0.515×VANGL2

The proportion of refractory patients increased progressively across SNP risk score quartiles, indicating a clear dose-effect relationship. Logistic regression analysis demonstrated that the SNP risk score was significantly associated with refractory hyperthyroidism (odds ratio [OR] = 2.7, 95% confidence interval [CI]: 1.8–4.1, *p* < 0.001).

### Early-therapy clinical prediction model

An early clinical prediction model for refractory GD was developed by integrating eight core clinical variables. These variables included five baseline indicators (age, baseline thyroid volume, baseline FT4, baseline TPOAb, and baseline TRAb) and three dynamic indicators at 3 months of therapy (relative dose-effect values of FT3, TRAb, and TPOAb). No significant interactions or multicollinearity were detected among the variables (VIF < 10).

Given the relatively balanced ratio of sample size to variable count, five modeling approaches were compared: logistic regression, ridge regression, random forest, extreme gradient boosting (XGBoost), and support vector machine (SVM). Candidate models were fitted or tuned using 10-fold cross-validation. In the development cohort, the random forest model showed the highest apparent area under the curve (AUC) among the five clinical models (AUC = 0.794, 95% CI: 0.733–0.855), with an accuracy of 0.722, sensitivity of 0.478, specificity of 0.903, and F1 score of 0.595 (Table 3; Figures 3A and 3B).

**Table 3:** Performance comparison of prediction models for refractory Graves’ disease (development cohort)

Model type	AUC	AUC 95% CI lower	AUC 95% CI upper	Accuracy	Sensitivity	Specificity	F1 score
**Clinical-onlymodel**							

Logistic	0.760	0.695	0.824	0.718	0.609	0.798	0.647
Ridge	0.760	0.695	0.825	0.704	0.522	0.839	0.600
Random forest	0.794	0.733	0.855	0.722	0.478	0.903	0.595
XGBoost	0.785	0.723	0.847	0.718	0.543	0.847	0.621
SVM	0.754	0.689	0.819	0.713	0.543	0.839	0.617

**Integrated model**							

Random forest (with candidate SNP risk score)	0.841	0.787	0.894	0.755	0.543	0.911	0.654

Performance metrics were calculated in the development cohort using a probability threshold of 0.50. AUC, area under the curve; CI, confidence interval; SVM, support vector machine; XGBoost, extreme gradient boosting.

**Figure 3:**
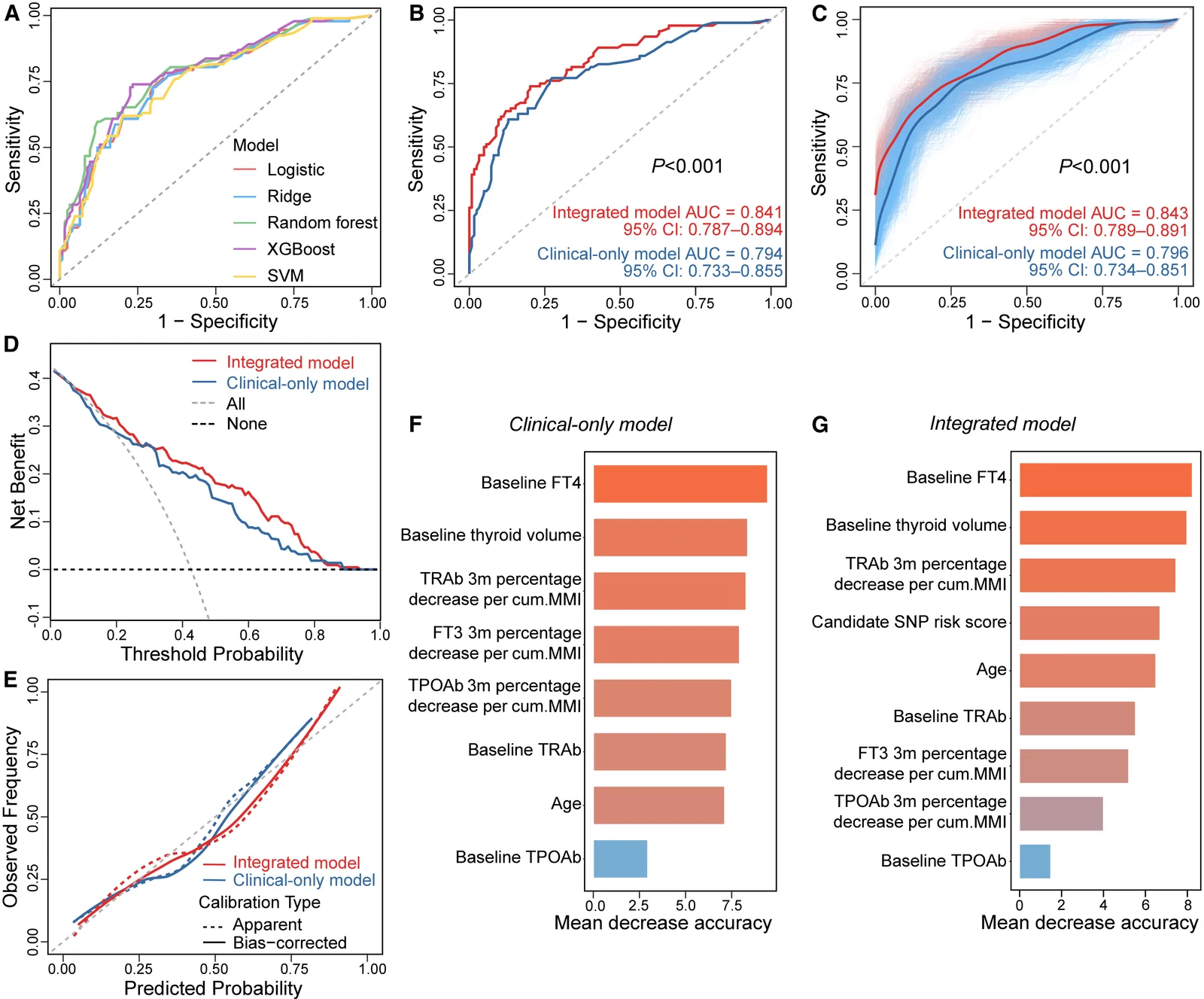
Performance of clinical-only and integrated random-forest models in the development cohort (*N* = 216) (A) Receiver operating characteristic (ROC) curves for five clinical models. (B) ROC curves for the clinical-only and integrated random forest models. (C) Bootstrap ROC curves (1000 resamples). (D) Decision curve analysis (DCA) curves. (E) Calibration plots. (F) Variable importance of the clinical-only model. (G) Variable importance of the integrated model. Variable importance is shown as mean decrease in accuracy. AUC, area under the curve; FT3, free triiodothyronine; FT4, free thyroxine; MMI, methimazole; m, month; SNP, single nucleotide polymorphism; TPOAb, thyroid peroxidase antibody; TRAb, thyroid-stimulating hormone receptor antibody.

The selected clinical model showed similar discrimination in the bootstrap resampling analysis (AUC = 0.796, 95% CI: 0.734–0.851) (Figure 3C). Calibration assessment showed a Brier score of 0.190, calibration intercept of 0.625, and calibration slope of 0.827 (Figure 3E). Variable importance analysis indicated that the baseline FT4 contributed strongly to the clinical model, followed by baseline thyroid volume and the relative dose-effect values of TRAb at 3 months of therapy (Figure 3F).

### Integrated prediction model of genetic polymorphisms and clinical characteristics

The predictive performance of the integrated model incorporating the candidate SNP risk score was significantly enhanced, with the AUC increasing from 0.794 to 0.841 (*p* < 0.001) (Table 3; Figure 3B). The integrated model demonstrated consistent performance in both the training set (AUC = 0.841, 95% CI: 0.787–0.894) and the internal bootstrap validation set (AUC = 0.843, 95% CI: 0.789–0.891) (Table 4; Figures 3B and 3C). Decision curve analysis showed that the integrated model provided higher net benefit than the clinical-only model across most threshold probabilities between 0.05 and 0.93, with the most continuous benefit observed over the clinically relevant range of approximately 0.32–0.82 (Figure 3D). Calibration assessment showed a Brier score of 0.168, calibration intercept of 0.505, and calibration slope of 1.227 (Figure 3E). Variable importance analysis showed that the leading contributors remained baseline FT4, baseline thyroid volume, and the relative dose-effect values of TRAb at 3 months of therapy, while the candidate SNP risk score ranked fourth in the integrated model (Figure 3G). Subgroup analyses of the two components of the composite refractory GD endpoint showed that the integrated model improved discrimination for post-withdrawal relapse in both cohorts, whereas its incremental discrimination for persistent MMI treatment after more than 2 years was not statistically significant (Table S12).

**Table 4:** TRIPOD-style performance of the selected random-forest models

Analysis	Cohort, *N* (events)	Model	AUC (95% CI)	Brier score	Calibration intercept	Calibration slope	ΔAUC *p* value
Apparent performance	development, 216 (92)	clinical	0.794 (0.733–0.855)	0.190	0.625	0.827	
		integrated	0.841 (0.787–0.894)	0.168	0.505	1.227	<0.001
Bootstrap resampling	development, 216 (92)	clinical	0.796 (0.734–0.851)	–	–	–	
		integrated	0.843 (0.789–0.891)	–	–	–	<0.001
Temporal validation	validation, 208 (125)	clinical	0.755 (0.687–0.824)	0.219	0.856	0.761	
		integrated	0.805 (0.744–0.866)	0.198	0.781	1.166	<0.001

Bootstrap resampling was performed with 1,000 resamples, using the selected random-forest models. Validation values were obtained from the temporal validation cohort. Calibration intercept and slope were estimated by logistic recalibration of observed outcomes on the logit-transformed predicted probability. The *p* value for ΔAUC compares the integrated model with the clinical model within each analysis. AUC, area under the curve; CI, confidence interval; TRIPOD, transparent reporting of a multivariable prediction model for individual prognosis or diagnosis.

Analysis of the risk reclassification metrics demonstrated that the integrated model showed modest but significant improvement in risk reclassification compared with the clinical-only model: category-based net reclassification improvement (NRI) = 0.133 (95% CI: 0.020–0.246, *p* = 0.022), and integrated discrimination improvement (IDI) = 0.033 (95% CI: 0.015–0.051, *p* < 0.001). Stratified analysis showed that 38 (17.6%) patients were reassigned to different risk strata, including 26 (12.0%) patients reclassified in the clinically correct direction and 12 (5.6%) patients reclassified in the incorrect direction. The greatest net benefit was observed in the intermediate-risk group, in which 20 of 64 (31.2%) patients were reclassified, including 17 (26.6%) in the correct direction and 3 (4.7%) in the incorrect direction (Table 5). These findings suggest that patients in the intermediate-risk category may derive the greatest benefit from SNP risk score testing. Threshold-specific operating characteristics of the clinical-only and integrated random-forest models in the development cohort at prespecified probabilities of 0.30, 0.50, and 0.60 are reported in Table S13.

**Table 5:** Risk reclassification analysis for refractory Graves’ disease

Cohort	Clinical-only risk stratum	*N* (events)	Reclassified	Correct direction	Incorrect direction	Net correct contribution
Development	low (<0.30)	113 (22)	9 (8.0%)	3 (2.7%)	6 (5.3%)	−0.027
	intermediate (0.30–0.59)	64 (39)	20 (31.2%)	17 (26.6%)	3 (4.7%)	0.219
	high (≥0.60)	39 (31)	9 (23.1%)	6 (15.4%)	3 (7.7%)	0.077
Temporal validation	low (<0.30)	61 (17)	7 (11.5%)	2 (3.3%)	5 (8.2%)	−0.049
	intermediate (0.30–0.59)	87 (61)	26 (29.9%)	21 (24.1%)	5 (5.7%)	0.184
	high (≥0.60)	60 (47)	16 (26.7%)	9 (15.0%)	7 (11.7%)	0.033

Risk strata were defined by the clinical-only model: low risk (<0.30), intermediate risk (0.30–0.59), and high risk (≥0.60). Reclassification refers to the movement to another risk stratum after adding the candidate SNP risk score. Upward movement was considered correct for patients with refractory Graves’ disease, whereas downward movement was considered correct for patients without refractory Graves’ disease. Net correct contribution was calculated as the proportion of correct reclassification minus the proportion of incorrect reclassification.

### Single-center temporal validation cohort

In the single-center temporal validation cohort, the predictive performance of the integrated model (AUC = 0.805, 95% CI: 0.744–0.866) was significantly higher than that of the clinical-only model (AUC = 0.755, 95% CI: 0.687–0.824; *p* < 0.001) (Table 4; Figure S1A). Decision curve analysis showed that the integrated model provided greater clinical net benefit than the clinical-only model across most clinically relevant threshold probabilities, with the most continuous benefit observed between approximately 0.32 and 0.73 (Figure S1B). Calibration assessment showed a Brier score of 0.198, calibration intercept of 0.781, and calibration slope of 1.166 for the integrated model (Figure S1C).

Reclassification analysis yielded consistent results, showing that the integrated model modestly but significantly improved risk stratification in the temporal validation cohort: category-based NRI = 0.152 (95% CI: 0.018–0.290, *p* = 0.030) and IDI = 0.027 (95% CI: 0.004–0.049, *p* = 0.021). A total of 49 (23.6%) patients were reassigned to different risk strata, including 32 (15.4%) reclassified in the clinically correct direction and 17 (8.2%) reclassified in the incorrect direction. The inter-mediate-risk group again showed the greatest incremental value, with the reclassification of 26 of 87 (29.9%) patients, including 21 (24.1%) in the correct direction and 5 (5.7%) in the incorrect direction (Table 5). These findings indicate that the integrated model maintained a modest but directionally consistent improvement in risk stratification in the single-center temporal validation cohort. Threshold-specific operating characteristics of the clinical-only and integrated models in the temporal validation cohort are reported in Table S13.

### Relationship between treatment duration and recurrence

In the development cohort, the median effective duration of ATD therapy was 753 days (interquartile range [IQR]: 535–1073 days), defined as completion of standardized treatment or continuation of therapy until January 2025 among patients who did not discontinue treatment prematurely. Cox regression analysis showed no significant linear association between treatment duration and the risk of hyperthyroidism recurrence (*p* = 0.641), even after adjustment for cumulative MMI and levothyroxine (LT_4_) doses, with no evidence of a linear trend (*p* = 0.628).

However, the restricted cubic spline (RCS) model indicated a potential nonlinear relationship (*p* = 0.057), revealing key inflection points: risk-benefit equilibrium at 753 days (≈2 years) and 1,098 days (≈3 years) and a peak recurrence risk at 934 days (≈2.5 years) (Figure 4A). Recurrence risk declined sharply after 1,106 days (≈3 years) (hazard ratio [HR] decreased by 0.32 per 100-day increase; *β* = −0.0032, *R*^*2*^ = 0.967) and stabilized after 1,385 days (≈3.8 years).

**Figure 4:**
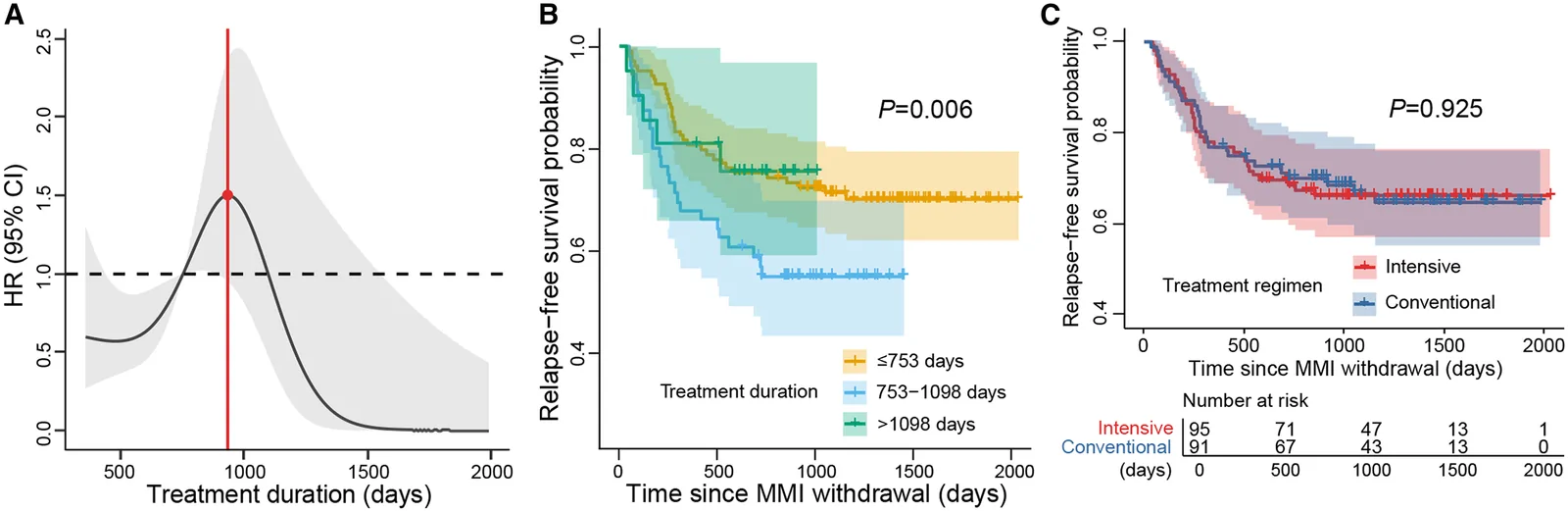
Relationship between ATD treatment duration and hyperthyroidism recurrence risk in the development cohort (*N* = 216) (A) Restricted cubic spline curve showing the relationship between ATD treatment duration and hyperthyroidism recurrence risk. (B) Kaplan-Meier curves for recurrence risk stratified by different ATD treatment durations. (C) Kaplan-Meier curves comparing recurrence risk between intensive and conventional ATD therapies. HR, hazard ratio.

Sensitivity analysis, stratified by treatment duration (≤753, 753–1,098, and >1,098 days), showed a significantly higher recurrence risk in the 2- to 3-year group compared with the ≤ 2-year group (HR = 2.00, 95% CI: 1.09–3.66, *p* = 0.025), whereas no difference was observed between the > 3-year and ≤ 2-year groups (*p* > 0.05). Patients treated for more than 3 years had a 69.5% lower recurrence risk than those treated for 2–3 years (*p* = 0.006) (Figure 4B). To exclude potential confounding by different ATD treatment regimens, further stratified analysis confirmed that the recurrence risk did not differ significantly between the two regimens across all treatment duration subgroups (all *p* > 0.05) (Figure 4C). However, residual confounding by disease severity, physician decision-making, patient preference, adherence, and follow-up patterns cannot be excluded.

### Sensitivity analysis of the candidate SNP risk score

To address whether the genetic signal was mainly driven by *CD40* rs1883832, we performed a sensitivity analysis comparing a *CD40*-only model with the four-SNP risk score model. When added to the selected clinical random forest model, the four-SNP score showed numerically higher discrimination than *CD40* alone in both the development cohort (AUC, 0.841 vs. 0.831; ΔAUC = 0.010, *p* = 0.251) and the validation cohort (AUC, 0.805 vs. 0.789; ΔAUC = 0.016, *p* = 0.085). However, these differences were not statistically significant (Table S14). These findings suggest that *CD40* rs1883832 contributed substantially to the genetic signal, while the four-SNP score provided only modest additional discrimination. Therefore, we refer to this genetic variable as a candidate SNP risk score rather than a polygenic risk score.

### Clinical implications for risk-stratified management

On the basis of the above findings, a stepwise risk-stratified management approach may be considered for patients with newly diagnosed GD. First, initial screening can be performed for refractory risk using baseline clinical characteristics and early-treatment response indicators. Second, patients identified as having intermediate risk by the clinical model may be offered SNP risk score testing, after which the integrated predictive model can be applied to refine risk stratification and improve assessment accuracy. Third, because the conventional dose-titration and modified partial block-and-replace regimens showed comparable efficacy and safety, conventional dose-titration remains a guideline-concordant first-line ATD strategy.^21^ Finally, for patients at high predicted risk or those who do not meet withdrawal criteria after 2 years of standardized therapy, continued ATD treatment beyond 3 years may be discussed in the context of disease activity, treatment tolerance, adherence, and patient preference, although this consideration is based on an observational association and has not yet been prospectively validated. For patients unwilling or unable to continue long-term medication or those with contraindications, definitive therapies such as RAI treatment or thyroidectomy may be considered earlier in the disease course.

## Discussion

Motivated by the need for early identification of refractory GD, we used a prospective randomized cohort for model development and a single-center temporal cohort for validation. To enable comparison of early responses across the treatment regimens, we applied ATD-LT4 dose correction and developed a dynamic prognostic model integrating clinical characteristics, dose-corrected early treatment responses, and a candidate SNP risk score to support risk stratification in GD.

As a secondary analysis of the randomized trial, the modified partial block-and-replace regimen did not improve refractory GD, post-withdrawal recurrence, or safety outcomes compared with conventional dose titration. Because the partial block-and-replace regimen requires concomitant MMI and LT4 and is more complex, with potential additional cost, the present findings do not support its routine use in newly diagnosed GD. This conclusion is consistent with the 2016 American Thyroid Association (ATA) guidelines—the latest comprehensive ATA guidance for hyperthyroidism and GD in nonpregnant adults—which recommend MMI followed by dose titration for most patients choosing ATD therapy and generally discourage block-and-replace therapy.^21^ Liver enzyme elevations and neutropenia in this cohort were predominantly mild and transient. In the treatment-emergent adverse events (Table S5), most recorded liver enzyme elevations were below 120 U/L, ALT exceeded 200 U/L in only one patient, and no patient had a neutrophil count below 1.0 × 10^9^/L. Meta-analyses indicate that liver enzyme elevations and mild neutropenia are relatively common laboratory abnormalities during ATD treatment for GD. These abnormalities are generally mild and transient and usually improve as hyperthyroidism is controlled.^22^^,^^23^ Accordingly, these predominantly mild events do not indicate an unexpected safety concern associated with either ATD regimen but reinforce the need for baseline blood-count and liver-profile assessment, symptom-based follow-up, and individualized dose adjustment during ATD therapy.

This study identified the core clinical predictors of refractory GD, encompassing two dimensions: baseline static characteristics and dynamic early-treatment response. At baseline, younger age, larger thyroid volume, and higher levels of FT4, TPOAb, and TRAb were significantly associated with refractory GD. Previous GD prognostic models, such as the GREAT score proposed by Vos et al.^24^ and the clinical activity score developed by Masiello et al.,^25^ have primarily focused on pre-treatment baseline characteristics. While these models provide valuable clinical references for predicting GD recurrence, they generally lack mechanisms for dynamic risk reassessment after treatment initiation, and few studies have specifically addressed the prediction of refractory GD, particularly in cases where withdrawal criteria are difficult to achieve.^26^^,^^27^^,^^28^ Our findings demonstrated that the relative dose-effect values of FT3, TRAb, and TPOAb at 3 months of therapy were the principal early clinical predictors of refractory GD. Because FT3 reflects circulating biologically active thyroid hormone, a low relative dose-effect value indicates poor early biochemical responsiveness to ATD exposure. As a GD-specific antibody, changes in TRAb levels directly reflect the intensity of humoral immune activity. A low relative dose-effect value indicates a slow decline in TRAb levels at a given ATD dose, suggesting persistent autoimmune dysregulation and an increased risk of refractory disease.^29^^,^^30^ In addition, changes in TPOAb levels reflect the degree of local autoimmune inflammation within the thyroid gland. A low relative dose-effect value suggests that ATD therapy has not effectively suppressed local inflammatory activity, resulting in ongoing immune-mediated tissue injury.^31^^,^^32^ These results confirm that dynamic variations in immunobiochemical indicators during early treatment allow a more precise assessment of therapeutic sensitivity than baseline static characteristics, thereby providing potential monitoring targets for early clinical risk intervention.

Genetic factors serve as key regulators of the heterogeneity in treatment response among patients with GD. From 13 GD-susceptible SNPs previously reported in East Asian populations, this study identified four loci—*CD40*, *HLA-DPB1*, *LPP*, and *VANGL2*—that were significantly associated with refractory GD. Each locus contributes to disease progression through distinct immunological mechanisms. *CD40* is involved in B cell activation and antibody production, and its polymorphisms may influence the generation of autoantibodies such as TRAb.^20^^,^^33^^,^^34^ *HLA-DPB1*, a gene belonging to the major histocompatibility complex class II family, participates in antigen presentation and immune regulation, thereby indirectly affecting the risk of refractory disease.^34^^,^^35^ *LPP* and *VANGL2* contribute to autoimmune dysregulation by modulating immune cell migration and signal transduction.^36^^,^^37^ Given the modest individual effects of single SNPs, constructing an SNP risk score by integrating multiple loci can more comprehensively capture the cumulative impact of genetic polymorphisms on immune response intensity, thus elucidating the genetic basis underlying inter-individual variation in ATD treatment response.

To enhance predictive accuracy, we incorporated the SNP risk score into the early-treatment clinical model and developed an integrated predictive framework combining genetic polymorphisms with dynamic clinical indicators. The AUC for this integrated model in both the training and validation cohorts was significantly higher than that of the clinical-only model, with moderate-risk patients identified as the subgroup deriving the greatest benefit from this approach. This model achieves complementarity between genetic and dynamic clinical monitoring dimensions, modestly improving the precision of early prediction. Notably, although the previously developed GREAT+ score for hyperthyroidism also integrates genetic markers, its foundational data were derived from European Caucasian populations.^24^ In contrast, the SNP loci included in the present study were selected based on data from Chinese and Japanese populations, as well as cross-ethnic GWAS, thereby improving its clinical applicability among East Asian patients.^17^^,^^18^^,^^19^^,^^20^

An exploratory nonlinear association between treatment duration and recurrence risk was observed. The RCS and duration-stratified analyses suggested that recurrence risk was significantly higher in the 2- to 3-year treatment group than in the ≤ 2-year group, whereas the risk declined markedly after 3 years of therapy and stabilized after approximately 3.8 years. Several previous studies have demonstrated that extending the duration of ATD therapy exerts a beneficial effect on hyperthyroidism prognosis. For instance, a 2019 RCT by Azizi et al. reported that the 4-year post-withdrawal recurrence rate was only 15% in patients receiving long-term MMI therapy for 60 months, significantly lower than the 51% observed in those receiving short-term therapy for 18 months.^38^ Similarly, Jin et al. confirmed the positive impact of prolonged ATD treatment: among 1,829 newly diagnosed patients followed-up for a median of 72.7 months, those receiving extended therapy achieved a remission rate of 55.6%, substantially higher than that observed in patients with shorter treatment courses, consistent with conventional therapy.^39^ Integrating the RCS model findings from this study with existing evidence, it may be inferred that for patients who do not meet withdrawal criteria within 2 years—such as those with persistently positive TRAb levels or unstable thyroid function—abrupt discontinuation or only modest treatment prolongation may increase the risk of recurrence. Conversely, extending therapy beyond 3 years may help reduce the recurrence risk and improve long-term prognosis. However, this finding does not overcome the inherent limitations of the nonrandomized treatment-duration analysis, and approximately 3 years should not be interpreted as a confirmed optimal cutoff or a universal treatment rule.

Based on a prospective randomized cohort, following ATD-LT4 dose equivalence correction, this study developed and validated a comprehensive predictive model for refractory Graves’ hyperthyroidism by integrating baseline clinical characteristics, dynamic dose-effect changes at 3 months of treatment, and a candidate SNP risk score based on GD-susceptible gene loci. The model exhibited favorable predictive performance in both the model development and validation cohorts. The study found no efficacy or safety advantage of the modified partial block-and-replace therapy over conventional dose titration. Therefore, it does not support replacing guideline-recommended dose titration with this more complex regimen. Additionally, a 3-year duration was identified as a potential treatment-duration reference point associated with lower recurrence risk, although this inference requires confirmation in prospective randomized studies. This study provides a risk-stratification tool developed in an East Asian cohort for the early and improved identification of high-risk patients with refractory GD who are prone to treatment failure, persistent disease, and post-withdrawal recurrence under standard therapy, as well as offers clinical evidence to guide dosing regimens and individualized treatment duration.

### Limitations of the study

These findings support a dynamic prognostic framework integrating baseline clinical characteristics, early treatment responses, and a candidate SNP risk score. Several limitations should be acknowledged. The single-center design may introduce selection bias and limit the external generalizability of the prediction models. Larger multicenter cohorts are needed to validate model performance across broader East Asian and other populations. At baseline, thyroid size was documented only as ultrasound-derived thyroid volume, whereas WHO clinical goiter grade was not consistently recorded, precluding direct comparison with the GREAT risk stratification model for predicting relapse in Graves’ hyperthyroidism. The prespecified RCT protocol defined TRAb of <1.5 IU/L as negative, consistent with our laboratory historical reporting standard. Although Roche recommends 1.75 IU/L as a diagnostic cutoff, neither threshold has been validated for treatment withdrawal.^40^ This difference may have affected the classification of patients with TRAb levels of 1.5–1.75 IU/L and limited the generalizability of our findings. Further studies are needed to establish and validate a reliable TRAb threshold for treatment withdrawal. Treatment duration was not randomized and may have been influenced by disease severity, treatment response, physician decision-making, adherence, and patient preference. Consequently, the observed association between treatment beyond approximately 3 years and lower recurrence risk remains exploratory and should not be interpreted as evidence of a causal benefit, a validated optimal cutoff, or a universal treatment rule. The ATD-LT4 correction coefficient lacks external validation; so, dose-corrected findings remain exploratory. Candidate SNPs were primarily selected from publicly available GWASs with relatively few GD cases, and GD-specific GWASs and family studies are needed to identify loci more directly associated with refractory GD. Dietary iodine intake, medication adherence, smoking intensity, Graves’ orbitopathy severity, vitamin D levels, psychosocial stress, and environmental exposures were not measured and may have contributed to residual variation in treatment response and recurrence.^41^^,^^42^^,^^43^ The feasibility, acceptability, and cost effectiveness of this risk-stratified implementation pathway, including targeted candidate SNP testing, were not formally assessed and require prospective evaluation before broader clinical use.

## Resource availability

### Lead contact

Requests for further information, resources, and reagents should be directed to the lead contact, Dr. Xuqin Zheng (zhengxuqin@njmu.edu.cn).

### Materials availability

No unique biological materials or in-house developed reagents were generated in this study. The customized improved multiplex ligase detection reaction (imLDR) SNP genotyping kit was commercially designed and synthesized by Genesky Biotechnologies. All other commercial kits, instruments, and drugs are described in the STAR Methods section and can be purchased from standard suppliers.

### Data and code availability

• All data reported in this paper will be shared by the lead contact upon request.
• The code used for constructing the predictive model is provided in the supplemental information.
• Any additional information required to reanalyze the data reported in this paper is available from the lead contact upon request.

## Acknowledgments

We thank Tiantian Li and Yue Li for the extraction of genomic DNA from blood samples. We also thank Xin Teng, Lekang Xu, Huiling Long, and other members of the research team for their contributions to clinical data collection and participant follow-up. This work was supported by the following grants: Key Project of Jiangsu Provincial Health Commission (K2024057) and Postgraduate Research & Practice Innovation Program of Jiangsu Province (SJCX25_0786 and SJCX25_0795).

## Author contributions

X.W., M.G., and J.W. contributed equally to this work, and the authorship order among these co-first authors was determined by their sequential contributions to data analysis, integrated modeling construction, and initial manuscript drafting; K.X. and Q.F. identified GD-susceptible genes from published literature and GWAS databases; Q.W., Y.C., Z.W., X.L., and X.X. collected clinical data and blood samples; T.Y. and X.Z. designed and supervised the entire study, and all authors participated in manuscript revision and formally approved the final version for submission.

## Declaration of interests

The authors declare that they have no competing interests.

## STAR★Methods

### Key resources table

**Table:** 

REAGENT or RESOURCE	SOURCE	IDENTIFIER
**Biologicalsamples**		

Fasting peripheral venous blood samples	The First Affiliated Hospital with Nanjing Medical University	N/A

**Chemicals,peptides,andrecombinantproteins**		

Methimazole	Merck	CAS: 60–56-0
Levothyroxine	Merck	CAS: 55–03-8
Exonuclease I	New England Biolabs	Cat#M0293S
Shrimp alkaline phosphatase	New England Biolabs	Cat#M0371S

**Criticalcommercialassays**		

ProCell (E170 system buffer)	Roche	Cat#11662988
CleanCell (E170 cleaning solution)	Roche	Cat#11662970
Blood genomic DNA extraction kit	Tiangen	Cat#DP348
ImLDR multiplex SNP genotyping kit	Genesky	N/A (customized)

**Software andalgorithms**		

R package 4.4.2	The R Project for Statistical Computing	https://www.r-project.org/
Prism 9.0	GraphPad	https://www.graphpad.com/
GeneMapper 4.1	Applied Biosystems	N/A

**Other**		

Automated electrochemiluminescence immunoassay system	Roche	Model: E170
Color Doppler ultrasound device	Siemens	N/A
NanoDrop spectrophotometer	Thermo Fisher	Model: ND-2000
ABI 3730xl Genetic Analyzer	Applied Biosystems (Thermo Fisher)	Cat#A41046

### Experimental model and subject participant details

#### Ethical statement

This study was designed as a secondary analysis based on a prospective cohort study. The study protocol was approved by the Ethics Committee of the First Affiliated Hospital of Nanjing Medical University (No. 2016SR037.A1). All procedures were conducted in accordance with the Declaration of Helsinki, complied with relevant local regulatory standards. The original trial was registered at ClinicalTrials.gov (NCT05461820). Written informed consent was obtained from all participants prior to enrollment.

#### Population

The study population comprised adult patients with newly diagnosed GD who were admitted to the Department of Endocrinology, First Affiliated Hospital with Nanjing Medical University. All participants were of East-Asian ancestry. Biological sex was recorded at baseline, and the age of enrolled subjects ranged from 18 to 65 years. Sociocultural gender-related variables were not collected in this study. The development cohort (*N* = 240) was a two-arm, open-label, prospective interventional trial in which participants were randomly assigned 1:1 to conventional or intensive therapy. The enrollment period for the development cohort was from October 2018 to December 2020. The validation cohort (*N* = 338) employed a prospective, observational design. Enrollment for this cohort occurred from January 2021 to May 2023.

### Method details

#### Inclusion and exclusion criteria

The study enrolled adult patients with newly diagnosed GD. Diagnosis of GD was confirmed by attending physicians based on three core criteria: (1) the presence of clinical symptoms or signs of hyperthyroidism; (2) a low TSH level (<0.270 mIU/L) with an elevated or normal FT4 level (>22.0 pmol/L); and (3) positive TRAb (>1.5 IU/L).^21^

Exclusion criteria included recent use of glucocorticoids or immunosuppressants; a history of thyroid surgery; the presence of other thyroid disorders, such as hyperfunctioning adenoma or subacute thyroiditis; malignant tumors or other autoimmune diseases; and pregnancy or lactation.

#### Study protocol

The development cohort, based on an interventional randomized trial, was divided into a conventional therapy group and an intensive therapy group. As a real-world observational cohort, all participants in the validation cohort received standard dose-titrated clinical treatment. MMI (Merck, Germany) was used as the ATD for all patients with GD. LT_4_ was prescribed to supplement thyroid hormone levels in cases of drug-induced hypothyroidism.

In the development cohort, patients were managed according to the original RCT protocol. MMI was initiated at 30 mg/day for patients with baseline FT4 ≥ 3 times the upper limit of normal, and 15 mg/day for those below this threshold. Thyroid function and autoantibodies were monitored every 4 weeks, and dose tapering was performed according to standardized criteria. In the conventional group, MMI was gradually tapered to a minimal dose, and withdrawal was considered after 12–18 months of treatment if euthyroidism was achieved and TRAb became negative. In the intensive group, MMI was tapered to 15 mg/day for maintenance, and LT_4_ was added as needed. After normalization of TPOAb, thyroglobulin antibody (TgAb), and TRAb, the 15 mg/day MMI maintenance regimen lasted 6 months before final tapering. For participants with persistently positive thyroid antibodies across all three markers after more than 2 years of treatment, the decision to extend therapy or initiate gradual dose reduction and withdrawal was made at the discretion of the treating physician, based on clinical judgment and the patient’s willingness to continue therapy. Detailed treatment protocols and dose adjustments are provided in the supplemental information.

#### Measurements

In this study, participants’ laboratory indicators and follow-up data were collected across three phases: baseline (pretreatment), the treatment period, and the post-withdrawal follow-up period. Baseline assessments were completed before treatment initiation and included demographic and clinical data—age, sex, smoking history, family history of thyroid disease, and ophthalmopathy evaluation—as well as laboratory and imaging parameters. These comprised thyroid function tests (FT3, FT4, TSH), thyroid autoantibodies (TPOAb, TgAb, TRAb), thyroid color Doppler ultrasonography, complete blood count, and liver and renal function tests. During treatment, follow-up visits were performed every 4–8 weeks to monitor thyroid function and thyroid autoantibody levels. After drug withdrawal, all patients were followed up for at least 1 year.

Serum concentrations of FT3, FT4, TSH, TPOAb, TgAb, and TRAb were measured using the MODULAR ANALYTICS E170 automated electrochemiluminescence immunoassay system and corresponding reagent kits (Roche Diagnostics, Germany). The reference ranges were as follows: FT3, 3.10–6.80 pmol/L; FT4, 12.00–22.00 pmol/L; TSH, 0.270–4.200 mIU/L; TPOAb, <34.0 IU/mL; TgAb, <115.0 IU/mL; and TRAb, <1.5 IU/L.

Thyroid volume was measured using a color Doppler ultrasound device (Siemens, Germany) equipped with a 5–15-MHz probe. The length (a), height (b), and thickness (c) (in millimeters) of the left and right thyroid lobes were recorded. Total thyroid volume (mL) was calculated as the sum of the left and right lobe volumes, where the volume of a single lobe (mL) = 0.479 × (a × b × c)/1000.^44^

#### Genotyping of SNPs

Fasting peripheral venous blood samples (2 mL) were collected from all participants at baseline using ethylenediaminetetraacetic acid (EDTA) anticoagulant tubes and stored at −80 °C prior to deoxyribonucleic acid (DNA) extraction. Genomic DNA was extracted from peripheral blood leukocytes using a blood genomic DNA extraction kit according to the manufacturer’s instructions (Tiangen). DNA concentration and purity were verified using a NanoDrop ND-2000 ultraviolet spectrophotometer (Thermo Fisher Scientific, USA), and DNA quality was assessed by 1% agarose gel electrophoresis.

Genotyping was performed using the improved multiplex ligase detection reaction (imLDR) multiplex SNP genotyping kit (Genesky). Target regions containing SNPs were amplified by polymerase chain reaction (PCR), and PCR products were purified using Exonuclease I and shrimp alkaline phosphatase (ExoI/SAP) and then used as templates for ligation reactions. Each SNP was detected by two 5′ allele-specific probes and one 3′ fluorescently labeled common probe in a single ligation reaction. Ligation products were separated by capillary electrophoresis on an ABI 3730xl Genetic Analyzer. Raw data were analyzed using GeneMapper 4.1 software (Applied Biosystems, USA).

A total of 13 GD-related SNPs were genotyped in the following order: rs13210649 (*CCR6*), rs1883832 (*CD40*), rs231779 (*CTLA4*), rs9319588 (*FBXL19-AS1*), rs1977710 (*FCRL2*), rs4947296 (*HCG22*), rs2281388 (*HLA-DPB1*), rs6457617 (*HLA-DQB1*), rs13136820 (*LINC02265*), rs6780858 (*LPP*), rs1521 (*MICA*), rs28414437 (*TSHR*), and rs117201373 (*VANGL2*). These loci were selected according to their reported associations with GD in GWAS and their distributions in East Asian populations (Table S15). Locus-specific PCR primers and ligation probes were designed for each SNP and are detailed in Table S16.

#### Outcomes and definitions

This study aimed to develop and validate an Asian-relevant clinical prediction model for refractory Graves’ hyperthyroidism, and to explore the effects of different ATD regimens and treatment durations on disease prognosis. Therefore, the primary outcome of this secondary analysis was the incidence of refractory Graves’ hyperthyroidism.

Refractory hyperthyroidism was defined as failure to achieve clinical drug withdrawal criteria following standardized ATD therapy or recurrence after meeting these criteria. Specifically, this condition included two scenarios: (1) patients who developed biochemical hyperthyroidism during the drug withdrawal phase and (2) patients with persistently positive TRAb who were unable to discontinue ATD therapy after more than 2 years of standardized treatment.^4^

#### Normalization of MMI and LT4 dosages

To achieve standardized quantification of doses in the combined MMI and LT_4_ treatment regimen, a simplified correction method based on the principle of pharmacological equivalence was established to normalize LT_4_ doses to the effective dose of MMI. Given that MMI and LT_4_ exert antagonistic effects on thyroid function indicators (FT3, FT4, and TSH), and considering that baseline FT4 level demonstrated the strongest correlation with outcomes in the treatment response prediction model, the baseline FT4 level was selected as the core variable for dose correction. A dose conversion formula was developed by introducing a correction coefficient k to calculate the adjusted MMI dose (Adj.MMI), as follows (Σ_3m_ represents the cumulative dose over 3 months of treatment):

$$\sum_{3m}\text{Adj}.\text{MMI}=\sum_{3m}\text{MMI}+k\times \sum_{3m}{\text{LT}}_{4}$$

During optimization of the *k* value, a grid search method (search range: −0.3 to −0.001) was applied to systematically evaluate the correlation between Adj.MMI and FT4 reduction under different *k* values. The optimal correction coefficient was determined to be *k* = −0.12. Validation results indicated that normalization using this coefficient effectively harmonized dosage measurements across patients. The Adj.MMI was significantly correlated with FT4 reduction (*r* = 0.478, *p* < 0.001), FT3 reduction (*r* = 0.420, *p* < 0.001), and TSH elevation (*r* = 0.211, *p* = 0.002), confirming a valid dose–effect relationship between the corrected dosage and thyroid function changes.

To verify the reliability of the correction method, a sensitivity analysis was conducted by comparing correlations between cumulative MMI dose—before and after correction—and changes in thyroid function indicators in the subgroup of patients who received early combined LT_4_ therapy. The results showed no statistically significant differences in any correlation coefficients before and after correction (Z-difference <1.96), indicating that the dose–effect relationships between adjusted MMI (Adj.MMI) and uncorrected MMI remained consistent. These findings confirmed the robustness of the correction coefficient (*k* = −0.12), demonstrating that the correction procedure did not alter the intrinsic correlation structure of the original data. The final standardized dose conversion formula was as follows:

$$\sum_{3m}\text{Adj}.\text{MMI}=\sum_{3m}\text{MMI}-0.12\times \sum_{3m}{\text{LT}}_{4}$$

### Quantification and statistical analysis

All statistical analyses were conducted using R software (version 4.4.2; R Foundation for Statistical Computing, Vienna, Austria). Graphs were generated using GraphPad Prism (version 9.0; GraphPad Software, San Diego, CA, USA). Continuous variables were expressed as mean ± standard deviation when normally distributed or as median (interquartile range) [*M* (*Q*_1_, *Q*_3_)] when non-normally distributed. Group comparisons were performed using *t*-tests or one-way analysis of variance for normally distributed variables and nonparametric tests for non-normally distributed variables. Categorical variables were compared using the chi-square test or Fisher’s exact test, while ordinal variables were analyzed using the rank-sum test. Missing predictor data were handled before model development using multiple imputation by chained equations under fully conditional specification. The development and validation cohorts were imputed separately, with twenty imputed datasets generated for each cohort. Each imputation chain was run for a maximum of 50 iteration cycles. Continuous variables were imputed using predictive mean matching. The conditional imputation models for categorical predictors incorporated interactions among categorical predictors. Pooled estimates were obtained according to Rubin’s rules. Genetic SNP variables were not subjected to imputation and were analyzed using the observed genotypes. The pre-imputation missingness and imputation strategy are reported in Table S17. Receiver operating characteristic curves were applied to determine optimal cutoff values for continuous variables.

The study population included a development cohort (per-protocol [PP] set from the 2018–2020 RCT) with internal validation using bootstrap resampling and a temporal observational validation cohort (2021–2023 non-RCT cohort from the same institution) to assess model generalizability. Refractory GD was the dependent variable. Baseline and 3-month treatment-response predictors were screened jointly. The clinical candidate set comprised 18 variables, including 12 baseline variables and 6 variables measured or derived at 3 months of therapy. Clinical predictors with *p* < 0.1 in univariable logistic regression were entered into LASSO regression with 10-fold cross-validation using the λ_1se_ criterion. Two standardized methods were employed to calculate 3-month drug dose–effect indicators: the absolute dose–effect value, defined as the absolute change in clinical parameters divided by the cumulative MMI dose, and the relative dose–effect value, defined as the percentage change in a clinical parameter divided by the adjusted cumulative MMI dose. The adjusted cumulative MMI dose was calculated using the ATD-LT4 dose-correction method described in Section “normalization of MMI and LT4 dosages”.

After integrating the core variables, 10-fold cross-validation was applied to compare five modeling approaches: logistic regression, ridge regression, random forest, XGBoost, and SVM. All candidate models were tuned using 10-fold cross-validation, with negative log loss as the selection metric. The tuning grids and selected hyperparameters are reported in Table S18. The random-forest model with the highest apparent AUC in the development cohort was selected as the clinical-only model. The selected random-forest model was evaluated using AUC with 95% confidence intervals, accuracy, sensitivity, specificity, F1 score, Brier score, calibration intercept and slope, decision-curve analysis, 1,000 bootstrap resamples, and temporal validation. To enhance predictive efficacy, genetic polymorphism variables were incorporated. Candidate SNPs were screened from 13 GD-susceptible loci using LASSO regression with 10-fold cross-validation (*λ*_min_ criterion). A candidate SNP risk score was then constructed by weighting each SNP’s regression coefficient (*β*, equivalent to log-OR) with its corresponding genotype value (0, 1, or 2, based on the number of risk alleles). The weighted candidate SNP risk score was categorized into five levels and incorporated into the selected clinical random-forest model. Clinical-only and integrated models were compared using discrimination, calibration, decision-curve analysis, category-based NRI, and IDI. Threshold-specific operating characteristics at predicted probabilities of 0.30, 0.50, and 0.60 are reported. A sensitivity analysis compared the integrated four-SNP model with a model including *CD40* rs1883832 alone.

#### Additional resources

This manuscript presents secondary analyses drawing on data from a study registered at ClinicalTrials.gov, identifier NCT05461820 (https://clinicaltrials.gov/ct2/show/NCT05461820).

## Supplemental information

Document S1. Figure S1 and Tables S1–S18

Data S1. Study design, treatment protocols, follow-up, and outcomes of the randomized controlled trial

Data S2. R code used for constructing the predictive model

## References

[1] Chaker L., Cooper D.S., Walsh J.P., Peeters R.P. (2024). Hyperthyroidism. Lancet.

[2] Hoang T.D., Stocker D.J., Chou E.L., Burch H.B. (2022). 2022 Update on Clinical Management of Graves Disease and Thyroid Eye Disease. Endocrinol. Metab. Clin. North Am..

[3] Villagelin D., Cooper D.S., Burch H.B. (2024). A 2023 International Survey of Clinical Practice Patterns in the Management of Graves Disease: A Decade of Change. J. Clin. Endocrinol. Metab..

[4] Wang X., Li T., Li Y., Wang Q., Cai Y., Wang Z., Shi Y., Yang T., Zheng X. (2024). Enhanced predictive validity of integrative models for refractory hyperthyroidism considering baseline and early therapy characteristics: a prospective cohort study. J. Transl. Med..

[5] Stancu A.M., Badiu C. (2024). Block-and-replace regimen versus titration of antithyroid drugs: a recent meta-analysis. Endokrynol. Pol..

[6] Lanzolla G., Marinò M., Menconi F. (2024). Graves disease: latest understanding of pathogenesis and treatment options. Nat. Rev. Endocrinol..

[7] Francis N., Francis T., Lazarus J.H., Okosieme O.E. (2020). Current controversies in the management of Graves' hyperthyroidism. Expert Rev. Endocrinol. Metab..

[8] Smolders A., Kyrilli A., Constantinescu S.M., Corvilain B., Daumerie C., Burlacu M.C. (2025). High-dose versus low-dose block-and-replace treatment for a first episode of Graves' disease. Eur. Thyroid J..

[9] Xiao S., Hu Y., Wang X., Yu H. (2025). Epigenetic Mechanisms in Autoimmune Thyroid Diseases: Bridging Research and Clinical Applications. Int. J. Mol. Sci..

[10] Mavridou M., Pearce S.H. (2025). Exploring antigenic variation in autoimmune endocrinopathy. Front. Immunol..

[11] Zhang W., Ding R., Hu Y., Wei W., Tian D., Qin N., Yu H., Wang X. (2024). Unraveling susceptibility genes: A contemporary overview of autoimmune thyroid diseases. Int. Immunopharmacol..

[12] Grixti L., Lane L.C., Pearce S.H. (2024). The genetics of Graves' disease. Rev. Endocr. Metab. Disord..

[13] Faustino L.C., Kahaly G.J., Frommer L., Concepcion E., Stefan-Lifshitz M., Tomer Y. (2021). Precision Medicine in Graves' Disease: CD40 Gene Variants Predict Clinical Response to an Anti-CD40 Monoclonal Antibody. Front. Endocrinol..

[14] Vejrazkova D., Vcelak J., Vaclavikova E., Vankova M., Zajickova K., Vrbikova J., Duskova M., Pacesova P., Novak Z., Bendlova B. (2021). Recurrence of Graves' Disease: What Genetics of HLA and PTPN22 Can Tell Us. Front. Endocrinol..

[15] Jansen H.I., Heuveling van Beek C., Bisschop P.H., Heijboer A.C., Bruinstroop E., Boelen A. (2024). The need for the GREAT+ score to predict relapse in Graves' disease: a questionnaire among patients and internal medicine specialists. J. Endocrinol. Investig..

[16] Langenstein C., Schork D., Badenhoop K., Herrmann E. (2016). Relapse prediction in Graves' disease: Towards mathematical modeling of clinical, immune and genetic markers. Rev. Endocr. Metab. Disord..

[17] Sakaue S., Kanai M., Tanigawa Y., Karjalainen J., Kurki M., Koshiba S., Narita A., Konuma T., Yamamoto K., Akiyama M. (2021). A cross-population atlas of genetic associations for 220 human phenotypes. Nat. Genet..

[18] Chu X., Pan C.M., Zhao S.X., Liang J., Gao G.Q., Zhang X.M., Yuan G.Y., Li C.G., Xue L.Q., Shen M. (2011). A genome-wide association study identifies two new risk loci for Graves' disease. Nat. Genet..

[19] Ishigaki K., Akiyama M., Kanai M., Takahashi A., Kawakami E., Sugishita H., Sakaue S., Matoba N., Low S.K., Okada Y. (2020). Large-scale genome-wide association study in a Japanese population identifies novel susceptibility loci across different diseases. Nat. Genet..

[20] Jiang H., Yuan F.F., Wang H.N., Liu W., Ye X.P., Yang S.Y., Xie H.J., Yu S.S., Ma Y.R., Zhang L.L. (2021). Compelling Evidence Linking CD40 Gene With Graves' Disease in the Chinese Han Population. Front. Endocrinol..

[21] Ross D.S., Burch H.B., Cooper D.S., Greenlee M.C., Laurberg P., Maia A.L., Rivkees S.A., Samuels M., Sosa J.A., Stan M.N., Walter M.A. (2016). 2016 American Thyroid Association Guidelines for Diagnosis and Management of Hyperthyroidism and Other Causes of Thyrotoxicosis. Thyroid.

[22] Scappaticcio L., Longo M., Maiorino M.I., Pernice V., Caruso P., Esposito K., Bellastella G. (2021). Abnormal Liver Blood Tests in Patients with Hyperthyroidism: Systematic Review and Meta-Analysis. Thyroid.

[23] Ramírez Stieben L.A., Brun L.R., Nasazzi Doddi P., Brance M.L. (2025). Antithyroid drugs and the dose-risk balance: a meta-analysis on agranulocytosis in hyperthyroidism. Endocrine.

[24] Vos X.G., Endert E., Zwinderman A.H., Tijssen J.G.P., Wiersinga W.M. (2016). Predicting the Risk of Recurrence Before the Start of Antithyroid Drug Therapy in Patients With Graves' Hyperthyroidism. J. Clin. Endocrinol. Metab..

[25] Masiello E., Veronesi G., Gallo D., Premoli P., Bianconi E., Rosetti S., Cusini C., Sabatino J., Ippolito S., Piantanida E. (2018). Antithyroid drug treatment for Graves' disease: baseline predictive models of relapse after treatment for a patient-tailored management. J. Endocrinol. Investig..

[26] El Kawkgi O., Toro-Tobon D., Toloza F.J.K., Vallejo S., Jacome C.S., Ayala I.N., Vallejo B.A., Wenczenovicz C., Tzeng O., Spencer H.J. (2025). A Predictive Model for Graves' Disease Recurrence After Antithyroid Drug Therapy: A Retrospective Multicenter Cohort Study. Endocrine practice. Endocr. Pract..

[27] Meling Stokland A.E., Austdal M., Nedrebø B.G., Carlsen S., Hetland H.B., Breivik L., Ueland H.O., Watt T., Cramon P.K., Løvås K. (2024). Outcomes of Patients With Graves Disease 25 Years After Initiating Antithyroid Drug Therapy. J. Clin. Endocrinol. Metab..

[28] Shah R., Adamson S.E., Jasim S. (2025). Management Aspects of Medical Therapy in Graves Disease. Endocrine practice. Endocr. Pract..

[29] Peng R., Xie P., Jin Z., Zhou W., Wang Y., Chen X., Yin Q., Shen L., Meng L., Ye L. (2025). Significance of Thyroid-Stimulating Immunoglobulin and Thyrotropin Receptor Antibody in Graves Disease. J. Clin. Endocrinol. Metab..

[30] Kim J., Choi M.S., Park J., Park H., Jang H.W., Choe J.-H., Kim J.-H., Kim J.S., Cho Y.S., Choi J.Y. (2021). Changes in Thyrotropin Receptor Antibody Levels Following Total Thyroidectomy or Radioiodine Therapy in Patients with Refractory Graves' Disease. Thyroid.

[31] Brancatella A., Torregrossa L., Viola N., Sgrò D., Casula M., Basolo F., Materazzi G., Marinò M., Marcocci C., Santini F., Latrofa F. (2023). In Graves' disease, thyroid autoantibodies and ultrasound features correlate with distinctive histological features. J. Endocrinol. Investig..

[32] Godlewska M., Banga P.J. (2019). Thyroid peroxidase as a dual active site enzyme: Focus on biosynthesis, hormonogenesis and thyroid disorders of autoimmunity and cancer. Biochimie.

[33] Bufalo N.E., Dos Santos R.B., Rocha A.G., Teodoro L., Romaldini J.H., Ward L.S. (2021). Polymorphisms of the genes CTLA4, PTPN22, CD40, and PPARG and their roles in Graves' disease: susceptibility and clinical features. Endocrine.

[34] Zhang Q.Y., Liu W., Li L., Du W.H., Zuo C.L., Ye X.P., Zhou Z., Yuan F.F., Ma Y.R., Sun F. (2020). Genetic Study in a Large Cohort Supported Different Pathogenesis of Graves' Disease and Hashimoto's Hypothyroidism. J. Clin. Endocrinol. Metab..

[35] Liao W.L., Liu T.Y., Cheng C.F., Chou Y.P., Wang T.Y., Chang Y.W., Chen S.Y., Tsai F.J. (2022). Analysis of HLA Variants and Graves' Disease and Its Comorbidities Using a High Resolution Imputation System to Examine Electronic Medical Health Records. Front. Endocrinol..

[36] Jiang H., Xie Y., Hu Z., Lu J., Zhang J., Li H., Zeng K., Peng W., Yang C., Huang J. (2025). VANGL2 alleviates inflammatory bowel disease by recruiting the ubiquitin ligase MARCH8 to limit NLRP3 inflammasome activation through OPTN-mediated selective autophagy. PLoS Biol..

[37] Sporkova A., Nahar T., Cao M., Ghosh S., Sens-Albert C., Friede P.A.P., Nagel A., Al-Hasani J., Hecker M. (2023). Characterisation of Lipoma-Preferred Partner as a Novel Mechanotransducer in Vascular Smooth Muscle Cells. Cells.

[38] Azizi F., Amouzegar A., Tohidi M., Hedayati M., Khalili D., Cheraghi L., Mehrabi Y., Takyar M. (2019). Increased Remission Rates After Long-Term Methimazole Therapy in Patients with Graves' Disease: Results of a Randomized Clinical Trial. Thyroid.

[39] Jin M., Jang A., Kim C.A., Kim T.Y., Kim W.B., Shong Y.K., Jeon M.J., Kim W.G. (2023). Long-term follow-up result of antithyroid drug treatment of Graves' hyperthyroidism in a large cohort. Eur. Thyroid J..

[40] Hermsen D., Broecker-Preuss M., Casati M., Mas J.C., Eckstein A., Gassner D., van Helden J., Inomata K., Jarausch J., Kratzsch J. (2009). Technical evaluation of the first fully automated assay for the detection of TSH receptor autoantibodies. Clin. Chim. Acta.

[41] Li S., Xu Q., Wang S., Peng H., Liu Y. (2025). Recent advances of trace elements in autoimmune thyroid disease. Front. Immunol..

[42] Piekiełko-Witkowska A., Duda M.K., Bogusławska J., Mackiewicz U. (2025). The impact of autoimmune thyroid disease on cognitive and psychiatric disorders: focus on clinical, pre-clinical and molecular studies. Eur. Thyroid J..

[43] Bjørklund G., Wallace D.R., Kangarlou K., Hossain F., Peana M. (2026). Environmental toxins and toxic metals in autoimmune diseases: Sex differences, hormonal influences, and immune dysregulation. Autoimmun. Rev..

[44] Brunn J., Block U., Ruf G., Bos I., Kunze W.P., Scriba P.C. (1981). [Volumetric analysis of thyroid lobes by real-time ultrasound (author's transl)]. Dtsch. Med. Wochenschr..

