# Supplementary material for: Genetic polymorphisms and early responses predict refractory hyperthyroidism during antithyroid drug therapy
Source: iScience. 2026 Oct 8;29(11):117780. doi: 10.1016/j.isci.2026.117780 (PMC13676917; doi:10.1016/j.isci.2026.117780)
Supplement: Supplementary file 1 [file mmc1.pdf]

iScience, Volume *29*

Supplemental information

Genetic polymorphisms and early responses predict refractory hyperthyroidism during antithyroid drug therapy

Xinpan Wang, Mengyi Gong, Jiayan Wang, Qiuyi Wang, Kuanfeng Xu, Qi Fu, Yun Cai, Zhixiao Wang, Xiaoyun Liu, Xinyu Xu, Tao Yang, and Xuqin Zheng

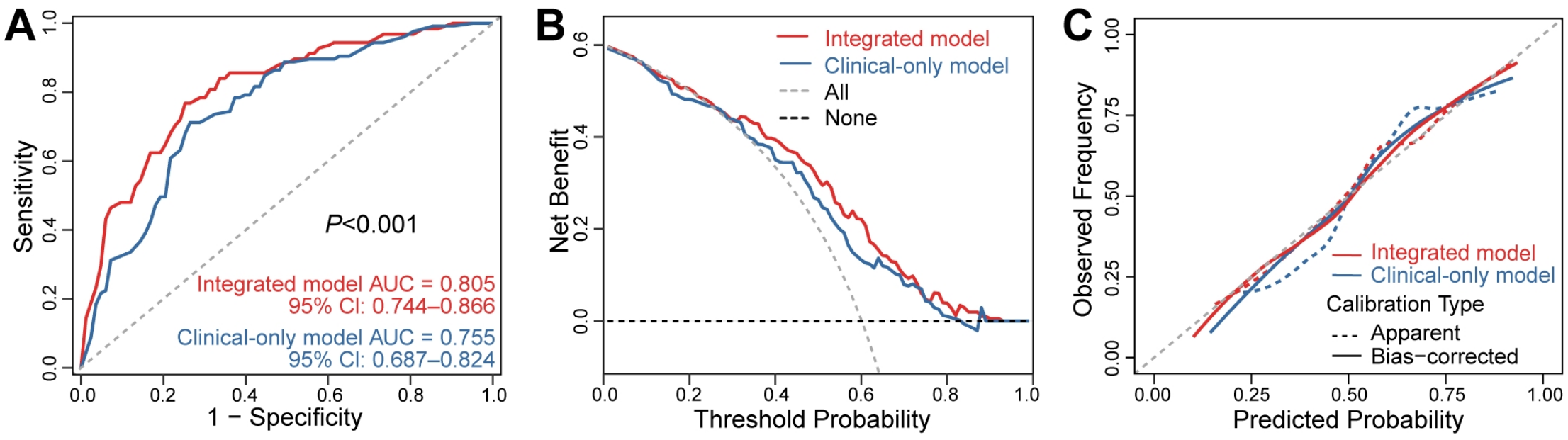

Figure S1 Performance of clinical-only and integrated random-forest models in the temporal validation cohort (*N*=208)
(A) Receiver operating characteristic (ROC) curves. (B) Decision curve analysis (DCA) curves. (C) Calibration plots. AUC, area under the curve.

Table S1 Baseline clinical characteristics of participants in the ITT analysis set of the RCT cohort

Baseline characteristics	Intensive (*N*=120)	Conventional (*N*=120)	*P* value
Age, y	37±12	35±10	0.146
Female, *n* (%)	95 (79.2%)	90 (75.0%)	0.443
Current smoking, *n* (%)	8 (6.7%)	14 (11.7%)	0.18
GD family history, *n* (%)	13 (10.8%)	12 (10.0%)	0.833
GO, *n* (%)	26 (21.7%)	24 (20.0%)	0.751
Baseline thyroid volume (cm^3)	16.9 (12.5, 22.2)	18.2 (13.0, 25.7)	0.086
Baseline FT3 (pmol/L)	22.4 (14.6, 35.2)	25.6 (18.1, 37.2)	0.086
Baseline FT4 (pmol/L)	60.6 (44.3, 87.0)	67.9 (49.2, 100)	0.083
Baseline TSH (mIU/L)	0.005 (0.005, 0.005)	0.005 (0.005, 0.005)	0.583
Baseline TPOAb (IU/mL)	103.4 (18.2, 293.5)	111.0 (26.9, 250.6)	0.797
Baseline TgAb (IU/mL)	158.3 (23.5, 407.8)	184.0 (28.0, 569.9)	0.292
Baseline TRAb (IU/L)	8.5 (3.8, 19.4)	9.7 (4.9, 20.4)	0.192

Continuous data are presented as mean ± SD or median (interquartile range, IQR) as appropriate. Categorical data are presented as *n* (%). FT3, free triiodothyronine; FT4, free thyroxine; GD, Graves' disease; GO, Graves' orbitopathy; ITT, intention to treat; RCT, randomized controlled trial; TgAb, thyroglobulin antibody; TPOAb, thyroid peroxidase antibody; TRAb, thyroid stimulating hormone receptor antibody; TSH, thyroid stimulating hormone.

Table S2 Baseline clinical characteristics of participants in the PP analysis set of the RCT cohort

Baseline characteristics	Intensive (*N*=109)	Conventional (*N*=107)	*P* value
Age, y	37±12	36±11	0.362
Female, *n* (%)	87 (79.8%)	82 (76.6%)	0.571
Current smoking, *n* (%)	7 (6.4%)	11 (10.3%)	0.305
GD family history, *n* (%)	11 (10.1%)	12 (11.2%)	0.789
GO, *n* (%)	22 (20.2%)	21 (19.6%)	0.918
Baseline thyroid volume (cm^3)	16.8 (12.7, 22.3)	17.6 (12.9, 25.1)	0.302
Baseline FT3 (pmol/L)	22.9 (15.4, 35.5)	23.9 (17.7, 36.0)	0.309
Baseline FT4 (pmol/L)	60.5 (44.3, 86.8)	64.3 (46.6, 100.0)	0.203
Baseline TSH (mIU/L)	0.005 (0.005, 0.005)	0.005 (0.005, 0.005)	0.25
Baseline TPOAb (IU/mL)	119.5 (20.1, 295.9)	88.9 (22.1, 214.0)	0.298
Baseline TgAb (IU/mL)	165.3 (24.3, 406.6)	152.7 (26.0, 560.1)	0.691
Baseline TRAb (IU/L)	8.8 (3.8, 19.3)	8.4 (4.3, 18.8)	0.611

Continuous data are presented as mean ± SD or median (interquartile range, IQR) as appropriate. Categorical data are presented as *n* (%). FT3, free triiodothyronine; FT4, free thyroxine; GD, Graves' disease; GO, Graves' orbitopathy; PP, per protocol; RCT, randomized controlled trial; TgAb, thyroglobulin antibody; TPOAb, thyroid peroxidase antibody; TRAb, thyroid stimulating hormone receptor antibody; TSH, thyroid stimulating hormone.

Table S3 Outcomes in the ITT analysis set of the RCT cohort

Outcome	Intensive (*N*=120)	Conventional (*N*=120)	*RR* (95% CI)	*P* value
Primary outcome, *n* (%)				
Refractory hyperthyroidism (failure to enter and sustain remission)	56 (46.7%)	57 (47.5%)	0.98 (0.75–1.28)	0.897
Secondary outcome, *n* (%)				
Post-withdrawal relapse	31 (25.8%)	32 (26.7%)	0.97 (0.63–1.48)	0.883
Persistent MMI treatment (>2 years, no withdrawal eligibility)	18 (15.0%)	13 (10.8%)	1.38 (0.71–2.70)	0.336

CI, confidence interval; ITT, intention to treat; MMI, methimazole; RCT, randomized controlled trial; RR, relative risk.

Table S4 Outcomes in the PP analysis set of the RCT cohort

Outcome	Intensive (*N*=109)	Conventional (*N*=107)	*RR* (95% CI)	*P* value
Primary outcome, *n* (%)				
Refractory hyperthyroidism (failure to enter and sustain remission)	48 (44.0%)	44 (41.1%)	1.07 (0.79–1.46)	0.665
Secondary outcome, *n* (%)				
Post-withdrawal relapse	30 (27.5%)	32 (29.9%)	0.92 (0.60–1.40)	0.699
Persistent MMI treatment (>2 years, no withdrawal eligibility)	18 (16.5%)	12 (11.2%)	1.47 (0.75–2.91)	0.260

CI, confidence interval; MMI, methimazole; PP, per protocol; RCT, randomized controlled trial; RR, relative risk.

Table S5 Treatment-emergent adverse events in the safety analysis set of the RCT cohort

	Intensive (*N*=120)	Conventional (*N*=120)	*RR* (95% CI)	*P* value
Liver enzyme elevation (ALT/AST, U/L), *n* (%)[a]				
<120	50 (41.7%)	46 (38.3%)	1.10 (0.82–1.47)	0.534
120–200	17 (14.2%)	15 (12.5%)	1.18 (0.64–2.18)	0.592
>200	0 (0.0%)	1 (0.8%)	—	1.000
Total	67 (55.8%)	62 (51.7%)	1.08 (0.85–1.37)	0.517
Neutropenia ($\times 10^9$/L), *n* (%)[b]				
1.5–2.0	22 (18.3%)	26 (21.7%)	0.85 (0.51–1.42)	0.539
1.0–1.5	5 (4.1%)	4 (3.3%)	1.20 (0.33–4.33)	1.000
Total	27 (22.5%)	30 (25.0%)	0.90 (0.57–1.42)	0.649
Rash, *n* (%)[c]				
Mild	8 (6.7%)	3 (2.5%)	2.60 (0.71–9.53)	0.133
Moderate-to-severe	8 (6.7%)	11 (9.2%)	0.76 (0.32–1.82)	0.536
Total	16 (13.3%)	14 (11.7%)	1.14 (0.58–2.24)	0.696
Other adverse events, *n* (%)				
Bilirubin elevation	7 (5.8%)	8 (6.7%)	0.88 (0.33–2.34)	0.790
Thrombocytopenia	1 (0.8%)	0 (0.0%)	—	1.000

[a]ALT normal reference range: 7–40 U/L. AST normal reference range: 13–35 U/L. Liver injury grading was mainly based on multiples of the upper limit of normal for ALT, with AST results as reference. [b]Normal reference range for neutrophils: 1.8–6.3$\times 10^9$/L; no patients in the RCT cohort had a neutrophil count < 1.0$\times 10^9$/L. [c]Severity grading of drug-induced rash followed Common Terminology Criteria for Adverse Events (CTCAE) Version 5.0. ALT: Alanine aminotransferase; AST: Aspartate aminotransferase; CI, confidence interval; RCT, randomized controlled trial; RR, relative risk.

Table S6 Genetic SNPs of patients in the refractory and non-refractory groups (development cohort)

SNP	Refractory (*N*=92)	Non-refractory (*N*=124)	*P* value
CCR6 rs13210649			0.912
T/T wildtype	23 (25.0%)	28 (22.6%)	
G/T heterozygote	48 (52.2%)	66 (53.2%)	
G/G homozygote	21 (22.8%)	30 (24.2%)	
CD40 rs1883832			<0.001
C/C wildtype	55 (59.8%)	40 (32.3%)	
C/T heterozygote	34 (37.0%)	68 (54.8%)	
T/T homozygote	3 (3.3%)	16 (12.9%)	
CTLA4 rs231779			0.506
T/T wildtype	53 (57.6%)	81 (65.3%)	
C/T heterozygote	34 (37.0%)	38 (30.6%)	
C/C homozygote	5 (5.4%)	5 (4.0%)	
FBXL19-AS1 rs9319588			0.499[a]
T/T wildtype	68 (73.9%)	100 (80.6%)	
C/T heterozygote	23 (25.0%)	23 (18.5%)	
C/C homozygote	1 (1.1%)	1 (0.8%)	
FCRL2 rs1977710			0.471
A/A wildtype	20 (21.7%)	36 (29.0%)	
A/G heterozygote	55 (59.8%)	66 (53.2%)	
G/G homozygote	17 (18.5%)	22 (17.7%)	
HCG22 rs4947296			0.292
T/T wildtype	55 (59.8%)	68 (54.8%)	
T/C heterozygote	30 (32.6%)	51 (41.1%)	
C/C homozygote	7 (7.6%)	5 (4.0%)	
HLA-DPB1 rs2281388			0.480
G/G wildtype	14 (15.2%)	22 (17.7%)	
G/A heterozygote	59 (64.1%)	84 (67.7%)	
A/A homozygote	19 (20.7%)	18 (14.5%)	
HLA-DQB1 rs6457617			0.664
T/T wildtype	35 (38.0%)	44 (35.5%)	
C/T heterozygote	43 (46.7%)	65 (52.4%)	
C/C homozygote	14 (15.2%)	15 (12.1%)	
LINC02265 rs13136820			0.731
T/T wildtype	16 (17.4%)	18 (14.5%)	
C/T heterozygote	45 (48.9%)	67 (54.0%)	
C/C homozygote	31 (33.7%)	39 (31.5%)	
LPP rs6780858			0.620
A/A wildtype	40 (43.5%)	47 (37.9%)	
A/G heterozygote	45 (48.9%)	64 (51.6%)	
G/G homozygote	7 (7.6%)	13 (10.5%)	
MICA rs1521			0.887[b]
T/T wildtype	80 (87.0%)	107 (86.3%)	
C/T heterozygote	12 (13.0%)	17 (13.7%)	
C/C homozygote	0 (0.0%)	0 (0.0%)	
TSHR rs28414437			0.808
C/C wildtype	41 (44.6%)	55 (44.4%)	
A/C heterozygote	43 (46.7%)	61 (49.2%)	
A/A homozygote	8 (8.7%)	8 (6.5%)	
VANGL2 rs117201373			0.147[a]
A/A wildtype	60 (65.2%)	95 (76.6%)	
A/G heterozygote	30 (32.6%)	27 (21.8%)	
G/G homozygote	2 (2.2%)	2 (1.6%)	

Wildtype, heterozygote and homozygote were defined according to genome-wide association study (GWAS) data from the East Asian normal population, with allele frequency references derived from the Allele Frequency Aggregator project (Release Version: 20250407153717) of the National Institutes of Health (NIH) database. [a]>20% of contingency table cells with expected frequency < 5; Fisher's exact test applied. [b]Two zero-frequency cells in the 3×2 table excluded; 2×2 Pearson's χ^2 test performed. SNP: single nucleotide polymorphism.

Table S7 Baseline clinical characteristics of patients in the refractory and non-refractory groups (validation cohort)

Baseline clinical characteristics	Refractory (*N*=125)	Non-refractory (*N*=83)	*P* value
Age, y	34±12	37±13	0.062
Female, *n* (%)	94 (75.2%)	65 (78.3%)	0.604
Baseline thyroid volume (cm^3)	26.0 (18.1, 35.8)	18.6 (15.0, 23.6)	<0.001
Baseline FT3 (pmol/L)	27.8 (19.7, 38.0)	22.8 (16.2, 33.6)	0.007
Baseline FT4 (pmol/L)	69.7 (52.5, 100.0)	60.3 (41.9, 75.8)	0.009
Baseline TSH (mIU/L)	0.005 (0.005, 0.005)	0.005 (0.005, 0.005)	0.615
Baseline TPOAb (IU/mL)	171.4 (44.6, 330.0)	126.5 (37.7, 290.8)	0.305
Baseline TgAb (IU/mL)	223.4 (41.0, 454.1)	220.9 (78.7, 492.8)	0.749
Baseline TRAb (IU/L)	21.7 (9.4, 30.9)	10.0 (7.1, 18.4)	<0.001

Continuous data are presented as mean ± SD or median (interquartile range, IQR) as appropriate. Categorical data are presented as *n* (%). FT3, free triiodothyronine; FT4, free thyroxine; TgAb, thyroglobulin antibody; TPOAb, thyroid peroxidase antibody; TRAb, thyroid stimulating hormone receptor antibody; TSH, thyroid stimulating hormone.

Table S8 Genetic SNPs of patients in the refractory and non-refractory groups (validation cohort)

SNP	Refractory (*N*=125)	Non-refractory (*N*=83)	*P* value
CCR6 rs13210649			0.192
T/T wildtype	34 (27.2%)	16 (19.3%)	
G/T heterozygote	58 (46.4%)	49 (59.0%)	
G/G homozygote	33 (26.4%)	18 (21.7%)	
CD40 rs1883832			<0.001
C/C wildtype	69 (55.2%)	25 (30.1%)	
C/T heterozygote	51 (40.8%)	43 (51.8%)	
T/T homozygote	5 (4.0%)	15 (18.1%)	
CTLA4 rs231779			1.000[a]
T/T wildtype	73 (58.4%)	48 (57.8%)	
C/T heterozygote	48 (38.4%)	32 (38.6%)	
C/C homozygote	4 (3.2%)	3 (3.6%)	
FBXL19-AS1 rs9319588			0.909[a]
T/T wildtype	111 (88.8%)	75 (90.4%)	
C/T heterozygote	13 (10.4%)	7 (8.4%)	
C/C homozygote	1 (0.8%)	1 (1.2%)	
FCRL2 rs1977710			0.472
A/A wildtype	42 (33.6%)	25 (30.1%)	
A/G heterozygote	59 (47.2%)	46 (55.4%)	
G/G homozygote	24 (19.2%)	12 (14.5%)	
HCG22 rs4947296			0.370
T/T wildtype	71 (56.8%)	52 (62.7%)	
T/C heterozygote	47 (37.6%)	24 (28.9%)	
C/C homozygote	7 (5.6%)	7 (8.4%)	
HLA-DPB1 rs2281388			0.529
G/G wildtype	22 (17.6%)	17 (20.5%)	
G/A heterozygote	81 (64.8%)	56 (67.5%)	
A/A homozygote	22 (17.6%)	10 (12.0%)	
HLA-DQB1 rs6457617			0.980
T/T wildtype	39 (31.2%)	27 (32.5%)	
C/T heterozygote	60 (48.0%)	39 (47.0%)	
C/C homozygote	26 (20.8%)	17 (20.5%)	
LINC02265 rs13136820			0.305
T/T wildtype	50 (40.0%)	29 (34.9%)	
C/T heterozygote	58 (46.4%)	36 (43.4%)	
C/C homozygote	17 (13.6%)	18 (21.7%)	
LPP rs6780858			0.530
A/A wildtype	53 (42.4%)	39 (47.0%)	
A/G heterozygote	62 (49.6%)	35 (42.2%)	
G/G homozygote	10 (8.0%)	9 (10.8%)	
MICA rs1521			0.112[a]
T/T wildtype	105 (84.0%)	63 (75.9%)	
C/T heterozygote	18 (14.4%)	20 (24.1%)	
C/C homozygote	2 (1.6%)	0 (0.0%)	
TSHR rs28414437			0.512[a]
C/C wildtype	69 (55.2%)	41 (49.4%)	
A/C heterozygote	53 (42.4%)	38 (45.8%)	
A/A homozygote	3 (2.4%)	4 (4.8%)	
VANGL2 rs117201373			0.077[a]
A/A wildtype	87 (69.6%)	67 (80.7%)	
A/G heterozygote	34 (27.2%)	12 (14.5%)	
G/G homozygote	4 (3.2%)	4 (4.8%)	

Wildtype, heterozygote and homozygote were defined according to genome-wide association study (GWAS) data from the East Asian normal population, with allele frequency references derived from the Allele Frequency Aggregator project (Release Version: 20250407153717) of the National Institutes of Health (NIH) database. [a]>20% of contingency table cells with expected frequency < 5; Fisher's exact test applied. SNP: single nucleotide polymorphism.

Table S9 Refractory odds ratios for baseline clinical characteristics in univariable analyses (development cohort)

Baseline clinical characteristics	Univariate analyses		
	Refractory, % (*n*/*N*)	OR (95% CI)	P value
Age, y			
>30	47/135 (34.8%)	Reference	
≤30	45/81 (55.6%)	2.3 (1.3–4.1)	0.003
Gender			
Female	72/169 (42.6%)	Reference	
Male	20/47 (42.6%)	1.0 (0.5–1.9)	0.995
Therapy group, *n* (%)			
Intensive	48/109 (44.0%)	1.1 (0.7–1.9)	0.665
Conventional	44/107 (41.1%)	Reference	
Current smoking, *n* (%)			
Yes	8/18 (44.4%)	1.1 (0.4–2.9)	0.868
No	84/198 (42.4%)	Reference	
GD family history, *n* (%)			
Yes	11/23 (47.8%)	1.3 (0.5–3.0)	0.592
No	81/193 (42.0%)	Reference	
GO, *n* (%)			
Yes	22/43 (51.2%)	1.5 (0.8–3.0)	0.206
No	70/143 (40.5%)	Reference	
Baseline thyroid volume (cm^3)			
> 28.0	18/24 (75.0%)	4.8 (1.8–12.6)	0.002
≤28.0	74/192 (38.5%)	Reference	
Baseline FT3 (pmol/L)			
> 25.0	53/99 (53.5%)	2.3 (1.3–4.0)	0.003
≤25.0	39/117 (33.3%)	Reference	
Baseline FT4 (pmol/L)			
>72.1	50/88 (56.8%)	2.7 (1.5–4.7)	<0.001
≤72.1	42/128 (32.8%)	Reference	
Baseline TPOAb (IU/mL)			
>170.9	45/85 (52.9%)	2.0 (1.2–3.5)	0.014
≤170.9	47/131 (35.9%)	Reference	
Baseline TgAb (IU/mL)			
>655.6	18/31 (58.1%)	2.1 (1.0–4.5)	0.063
≤655.6	74/185 (40.0%)	Reference	
Baseline TRAb (IU/L)			
>20.6	27/45 (60.0%)	2.4 (1.2–4.8)	0.009
≤20.6	65/171 (38.0%)	Reference	

CI, confidence interval; FT3, free triiodothyronine; FT4, free thyroxine; GD, Graves' disease; GO, Graves' orbitopathy; OR, Odds Ratio; TgAb, thyroglobulin antibody; TPOAb, thyroid peroxidase antibody; TRAb, thyroid stimulating hormone receptor antibody; TSH, thyroid stimulating hormone.

Table S10 Comparison of thyroid function and autoantibody indicators of the patients at 3 months of therapy

Characteristics of early therapy	Refractory (*N*=92)	Non-refractory (*N*=124)	*P* value
FT3			
3m value (pmol/L)	4.3 (3.6, 5.8)	4.3 (3.7, 5.1)	0.687
3m decrease (pmol/L)	21.4 (13.1, 32.6)	17.4 (10.5, 26.1)	0.025
3m percentage decrease (%)	77.5±14.5	74.8±16.0	0.216
3m decrease per adj.cum.MMI (pmol/L·g⁻¹)	13.2 (8.6, 19.5)	12.8 (8.3, 18.4)	0.561
3m percentage decrease per adj.cum.MMI (%·g⁻¹)	47.8 (37.7, 63.6)	53.8 (43.6, 68.7)	0.041
FT4			
3m value (pmol/L)	12.9 (9.2, 17.6)	12.4 (10.0, 14.6)	0.852
3m decrease (pmol/L)	59.0 (31.4, 84.4)	44.3 (28.5, 65.3)	0.020
3m percentage decrease (%)	75.9±17.7	73.8±16.5	0.368
3m decrease per adj.cum.MMI (pmol/L·g⁻¹)	34.0 (23.6, 45.8)	32.9 (21.7, 43.7)	0.489
3m percentage decrease per adj.cum.MMI (%·g⁻¹)	46.8 (36.1, 61.8)	52.9 (42.7, 65.6)	0.035
TSH			
3m value (mIU/L)	0.12 (0.01, 3.60)	0.91 (0.01, 5.00)	0.176
3m increase (mIU/L)	0.10 (0.00, 3.59)	0.90 (0.00, 5.00)	0.161
3m percentage increase (%)	2070.0 (0.0, 65850.0)	18050.0 (80.0, 89935.7)	0.105
3m increase per adj.cum.MMI (nIU/L·g⁻¹)	54.1 (0.0, 2613.9)	804.9 (3.0, 3431.2)	0.086
3m percentage increase per adj.cum.MMI (%·mg⁻¹)	1.1 (0.0, 47.1)	16.1 (0.1, 64.9)	0.053
TPOAb			
3m value (IU/mL)	121.2 (23.6, 255.7)	55.8 (16.0, 180.7)	0.039
3m decrease (IU/mL)	11.3 (-0.4, 49.6)	10.9 (-0.1, 51.8)	0.918
3m percentage decrease (%)	17.1 (-4.3, 30.3)	25.4 (-0.7, 35.4)	0.105
3m decrease per cum.MMI (IU/L·mg⁻¹)	7.6 (-0.3, 30.0)	7.0 (-0.1, 38.2)	0.641
3m percentage decrease per cum.MMI (%·g⁻¹)	9.8 (-2.8, 19.1)	14.8 (-0.5, 28.3)	0.066
TgAb			
3m value (IU/mL)	48.8 (15.3, 305.8)	69.9 (14.7, 286.5)	0.990
3m decrease (IU/mL)	23.0 (0.0, 150.4)	22.1 (0.0, 166.4)	0.904
3m percentage decrease (%)	29.8 (0.0, 56.6)	26.0 (0.0, 60.0)	0.847
3m decrease per cum.MMI (IU/L·mg⁻¹)	10.7 (0.0, 100.2)	20.4 (0.0, 122.9)	0.956
3m percentage decrease per cum.MMI (%·g⁻¹)	15.7 (0.0, 36.9)	16.7 (0.0, 44.0)	0.682
TRAb			
3m value (IU/L)	6.9 (2.9, 17.4)	4.6 (2.3, 9.3)	0.042
3m decrease (IU/L)	2.2 (0.7, 6.4)	2.4 (0.8, 6.0)	0.730
3m percentage decrease (%)	33.5 (12.0, 51.6)	41.0 (23.4, 55.6)	0.073
3m decrease per cum.MMI (IU/L·g⁻¹)	1.4 (0.4, 4.0)	2.3 (0.6, 4.4)	0.233
3m percentage decrease per cum.MMI (%·g⁻¹)	20.4 (6.2, 31.5)	28.3 (14.9, 42.5)	0.005
3m cum.MMI (mg)	1448 (1276, 2151)	1380 (1133, 1693)	0.021
3m cum.LT_4 (μg)	0 (0, 0)	0 (0, 0)	0.783
3m adj.cum.MMI (mg)	1415 (1220, 2148)	1363 (1096, 1693)	0.034

FT3, free triiodothyronine; FT4, free thyroxine; LT_4, Levothyroxine; MMI, Methimazole; TgAb, thyroglobulin antibody; TPOAb, thyroid peroxidase antibody; TRAb, thyroid stimulating hormone receptor antibody; TSH, thyroid stimulating hormone.

Table S11 Univariate analysis of early drug response (dose–effect values)

Characteristics of early therapy	Univariate analyses		
	Refractory, % (*n*/*N*)	*OR* (95% CI)	*P* value
FT3–3m decrease per adj.cum.MMI (pmol/L·g⁻¹)			
>21.8	57.6 (19/33)	2.0 (1.0–4.3)	0.062
≤21.8	39.9 (73/183)	Reference	
FT3–3m percentage decrease per adj.cum.MMI (%·g⁻¹)			
>45.4	35.9 (51/142)	Reference	
≤45.4	55.4 (41/74)	2.2 (1.3–3.9)	0.006
FT4–3m decrease per adj.cum.MMI (pmol/L·g⁻¹)			
>51.7	54.3 (19/35)	1.8 (0.8–3.6)	0.129
≤51.7	40.3 (73/181)	Reference	
FT4–3m percentage decrease per adj.cum.MMI (%·g⁻¹)			
>47.4	35.2 (44/125)	Reference	
≤47.4	52.7 (48/91)	2.1 (1.2–3.6)	0.010
TSH–3m increase per adj.cum.MMI (nIU/L·g⁻¹)			
>23.4	35.9 (46/128)	Reference	
≤23.4	52.3 (46/88)	2.0 (1.1–3.4)	0.018
TSH–3m percentage increase per adj.cum.MMI (%·mg⁻¹)			
>14.8	34.7 (34/98)	Reference	
≤14.8	49.2 (58/118)	1.8 (1.0–3.2)	0.033
TPOAb–3m decrease per cum.MMI (IU/L·mg⁻¹)			
>24.8	35.8 (24/67)	Reference	
≤24.8	45.6 (68/149)	1.5 (0.8–2.7)	0.178
TPOAb–3m percentage decrease per cum.MMI (%·g⁻¹)			
>22.8	25.8 (16/62)	Reference	
≤22.8	49.4 (76/154)	2.8 (1.5–5.4)	0.002
TgAb–3m decrease per cum.MMI (IU/L·mg⁻¹)			
>128.8	34.8 (16/46)	Reference	
≤128.8	44.7 (76/170)	1.5 (0.8–3.0)	0.229
TgAb–3m percentage decrease per cum.MMI (%·g⁻¹)			
>46.6	29.3 (12/41)	Reference	
≤46.6	45.7 (80/175)	2.0 (1.0–4.2)	0.058
TRAb–3m decrease per cum.MMI (IU/L·g⁻¹)			
>2.1	34.7 (35/101)	Reference	
≤2.1	49.6 (57/115)	1.9 (1.1–3.2)	0.028
TRAb–3m percentage decrease per cum.MMI (%·g⁻¹)			
>26.6	28.4 (27/95)	Reference	
≤26.6	53.7 (65/121)	2.9 (1.7–5.2)	<0.001

CI, confidence interval; FT3, free triiodothyronine; FT4, free thyroxine; MMI, Methimazole; OR, Odds Ratio; TgAb, thyroglobulin antibody; TPOAb, thyroid peroxidase antibody; TRAb, thyroid stimulating hormone receptor antibody; TSH, thyroid stimulating hormone.

Table S12 Subgroup analyses of endpoint components in the development and validation cohorts

Cohort	Endpoint	Events	Clinical-only model AUC	Integrated model AUC	*P* value
Development cohort					
Withdrawal (*N*=186)	Relapse	62 (33.3%)	0.741 (0.664–0.818)	0.8 (0.732–0.869)	<0.001
Therapy duration >2 years (*N*=114)	Persistent MMI treatment	30 (26.3%)	0.806 (0.720–0.891)	0.821 (0.738–0.904)	0.372
Validation cohort					
Withdrawal (*N*=191)	Relapse	97 (50.8%)	0.712 (0.639–0.786)	0.757 (0.689–0.826)	0.003
Therapy duration >2 years (*N*=110)	Persistent MMI treatment	28 (25.5%)	0.709 (0.600–0.818)	0.726 (0.623–0.830)	0.527

Clinical-only model: comprised of baseline clinical characteristics and early-treatment clinical characteristics. Integrated model: comprised of baseline clinical characteristics, early-treatment clinical characteristics, and candidate SNP risk score. AUC, area under the curve; MMI, methimazole.

Table S13 Threshold-specific performance of the selected random-forest models

Model setting and threshold	TP	FP	TN	FN	Accuracy	Sensitivity	Specificity	PPV	NPV	F1
Development (clinical-only)										
0.30	70	33	91	22	0.745	0.761	0.734	0.680	0.805	0.718
0.50	44	12	112	48	0.722	0.478	0.903	0.786	0.700	0.595
0.60	31	8	116	61	0.681	0.337	0.935	0.795	0.655	0.473
Development (integrated)										
0.30	70	32	92	22	0.750	0.761	0.742	0.686	0.807	0.722
0.50	50	11	113	42	0.755	0.543	0.911	0.820	0.729	0.654
0.60	38	2	122	54	0.741	0.413	0.984	0.950	0.693	0.576
Validation (clinical-only)										
0.30	108	39	44	17	0.731	0.864	0.530	0.735	0.721	0.794
0.50	73	18	65	52	0.663	0.584	0.783	0.802	0.556	0.676
0.60	47	13	70	78	0.562	0.376	0.843	0.783	0.473	0.508
Validation (integrated)										
0.30	107	38	45	18	0.731	0.856	0.542	0.738	0.714	0.793
0.50	81	17	66	44	0.707	0.648	0.795	0.827	0.600	0.726
0.60	55	6	77	70	0.635	0.440	0.928	0.902	0.524	0.591

Thresholds were prespecified for reporting to show the operating characteristics across clinically interpretable risk cut points. FN, false negative; FP, false positive; NPV, negative predictive value; PPV, positive predictive value; TN, true negative; TP, true positive.

Table S14 Comparison of *CD40* SNP and candidate four-SNP risk score models added to the selected clinical random-forest model

Model	AUC (95% CI)	Brier score	Accuracy	Sensitivity	Specificity	F1
Development						
Clinical-only	0.794 (0.733-0.855)	0.19	0.722	0.478	0.903	0.595
Integrated (Clinical + *CD40* SNP)	0.831 (0.775-0.886)	0.172	0.741	0.522	0.903	0.632
Integrated (Clinical + four-SNP score)	0.841 (0.787-0.894)	0.168	0.755	0.543	0.911	0.654
Validation						
Clinical-only	0.755 (0.687-0.824)	0.219	0.663	0.584	0.783	0.676
Integrated (Clinical + *CD40* SNP)	0.789 (0.726-0.853)	0.202	0.688	0.624	0.783	0.706
Integrated (Clinical + four-SNP score)	0.805 (0.744-0.866)	0.198	0.707	0.648	0.795	0.726

CD40 rs1883832 was modeled as a dosage factor coded 0, 1, and 2 according to the number of risk alleles. The candidate four-SNP risk score included *CD40* rs1883832, *HLA-DPB1* rs2281388, *LPP* rs6780858, and *VANGL2* rs117201373 and was modeled as a five-level categorical variable. Operating characteristics were calculated at a probability threshold of 0.50. AUC, area under the curve; CI, confidence interval; SNP: single nucleotide polymorphism.

Table S15 Information of 13 GD-associated SNPs: GWAS significance, effect sizes and ethnic distributions

SNP ID	Closest Gene	*P*-value	*OR*	95% CI	Ethnic Group
rs13210649	*CCR6*	7.00E-10	–	0.092–0.18	Transethnic[a]
rs1883832	*CD40*	9.77E-03	1.15	1.03–1.28	Chinese (East Asian)
rs231779	*CTLA4*	4.00E-26	–	0.19–0.28	Transethnic
rs9319588	*FBXL19-AS1*	1.00E-08	0.74	0.66–0.82	Japanese (East Asian)
rs1977710	*FCRL2*	5.00E-11	–	0.10–0.19	Transethnic
rs4947296	*HCG22*	4.00E-51	1.77	1.60–1.90	Chinese (East Asian)
rs2281388	*HLA-DPB1*	2.00E-65	1.64	1.50–1.70	Chinese (East Asian)
rs6457617	*HLA-DQB1*	7.00E-33	1.40	1.30–1.50	Chinese (East Asian)
rs13136820	*LINC02265*	2.00E-08	–	-0.18– -0.089	Transethnic
rs6780858	*LPP*	4.00E-13	–	-0.21– -0.12	Transethnic
rs1521	*MICA*	2.00E-65	1.92	1.80–2.10	Chinese (East Asian)
rs28414437	*TSHR*	2.00E-36	–	0.24–0.33	Transethnic
rs117201373	*VANGL2*	3.00E-13	1.60	1.40–1.80	Japanese (East Asian)

[a]Transethnic refers to Japanese (East Asian) and European (Caucasian) populations. CI, confidence interval; GD, Graves' disease; GWAS, genome-wide association study; OR, odds ratio; SNP, single nucleotide polymorphism.

Table S16 Locus-specific primers used for multiplex SNP genotyping by imLDR assay

SNP ID	Primer Name	Sequence (5'→3')
rs1883832	rs1883832_TF	TGTTCGTGGGCCGGATTAGTCCGCCTGGTCTCACCTCTCT
	rs1883832_CF	TGTTCGTGGGCCGGATTAGTTTCCGCCTGGTCTCACCTCTCC
	rs1883832_FP	ATGGTTCGTCTGCCTCTGCATTTTTTTTT
rs2281388	rs2281388_GR	TTCCGCGTTCGGACTGATATTCTGGGCCAGCCCAACAACC
	rs2281388_AR	TTCCGCGTTCGGACTGATATTTTCTGGGCCAGCCCAACAGCT
	rs2281388_RP	TCGTCTGCCATGTTGACAAGTTCTTTTTTTTT
rs28414437	rs28414437_AF	TACGGTTATTCGGGCTCCTGatccacccacctcggccAca
	rs28414437_CF	TACGGTTATTCGGGCTCCTGTTatccacccacctcggccAcC
	rs28414437_FP	caaaatgctgagattacaggcatgaTTTTTTT
rs1977710	rs1977710_AR	TCTCTCGGGTCAATTCGTCCtcaggtgatctgccacctagTct
	rs1977710_GR	TCTCTCGGGTCAATTCGTCCTTtcaggtgatctgccacctagTcC
	rs1977710_RP	tccaaaaStgctgagattacaggcTTTTT
rs1521	rs1521_CF	TGTTCGTGGGCCGGATTAGTaggcctggtgtgaatgattctcTgc
	rs1521_TF	TGTTCGTGGGCCGGATTAGTTTaggcctggtgtgaatgattctcTgT
	rs1521_FP	tcaggatttcacgaagctgtgtcTTTTTT
rs9319588	rs9319588_CF	TTCCGCGTTCGGACTGATATCACGGTCCTGGACTAGATTTCAGTAC
	rs9319588_TF	TTCCGCGTTCGGACTGATATTTCACGGTCCTGGACTAGATTTCAGCAT
	rs9319588_FP	TTTTCAAGAGAAGCTGGAAATCCATTTTTTT
rs13136820	rs13136820_CR	TACGGTTATTCGGGCTCCTGCGTTGGTCCCTTGGAAAACG
	rs13136820_TR	TACGGTTATTCGGGCTCCTGTTCGTTGGTCCCTTGGAAAGCA
	rs13136820_RP	GAACAGTACCAACCAAGAAGTTTCTACTTCTTTTTTT
rs231779	rs231779_CF	TCTCTCGGGTCAATTCGTCCCAGCCACTATTTTTGAGTTGATGCATGC
	rs231779_TF	TCTCTCGGGTCAATTCGTCCTTCAGCCACTATTTTTGAGTTGATGCACGT
	rs231779_FP	CTCTCTGTATGGAGAGCTGGTCTCCTTTT
rs13210649	rs13210649_TF	TGTTCGTGGGCCGGATTAGTagaggtacatagggaaagatccaaagTgt
	rs13210649_GF	TGTTCGTGGGCCGGATTAGTTTagaggtacatagggaaagatccaaagTgG
	rs13210649_FP	tcctgagcgtaagagttcctgtcttTTTTT
rs6457617	rs6457617_CF	TTCCGCGTTCGGACTGATATTCCCCATATGCACAGATCTTTGTTAGTGAC
	rs6457617_TF	TTCCGCGTTCGGACTGATATTTTCCCCATATGCACAGATCTTTGTTAGTGAT
	rs6457617_FP	ATCTGCTCATGGACTCAACAARCATTTTTTTT
rs4947296	rs4947296_TR	TACGGTTATTCGGGCTCCTGTCACTGTTTCCATTCACTAGTTTCCATATTGCA
	rs4947296_CR	TACGGTTATTCGGGCTCCTGTTTCACTGTTTCCATTCACTAGTTTCCATATTACG
	rs4947296_RP	TTGYGTCCTTTTTTGCTCTTGTTTTTTTT
rs6780858	rs6780858_AR	TCTCTCGGGTCAATTCGTCCgttcctgtgagcatttcctttgagAat
	rs6780858_GR	TCTCTCGGGTCAATTCGTCCTTgttcctgtgagcatttcctttgagAaC
	rs6780858_RP	tgtgttagcactcagaaagtttcagatttTTTTTT
rs117201373	rs117201373_AR	TGTTCGTGGGCCGGATTAGTagtacactgtgatttattcacagaacggaatatGat
	rs117201373_GR	TGTTCGTGGGCCGGATTAGTTTagtacactgtgatttattcacagaacggaatatAaC
	rs117201373_RP	acaacaacaataaaaaYtataggccaggc

Uppercase letters indicate target-specific complementary sequences; lowercase letters represent linker/spacer sequences. Degenerate bases: Y=C/T, R=A/G, K=G/T, M=A/C. imLDR, improved multiplex ligase detection reaction; SNP, single nucleotide polymorphism.

Table S17 Missing data before multiple imputation and candidate clinical predictors used for model development

Candidate predictor	Variable type	Missing before MI, n (%)		Conditional imputation model	Retained in final model
		Development cohort (*N*=216)	Validation cohort (*N*=208)		
Age	Continuous	0	0	—	Yes
Female	Binary	0	0	—	No
Baseline thyroid volume	Continuous	11 (5.1%)	10 (4.8%)	PMM	Yes
Current smoking	Binary	5 (2.3%)	9 (4.3%)	Categorical FCS model	No
GD family history	Binary	5 (2.3%)	9 (4.3%)	Categorical FCS model	No
GO	Binary	5 (2.3%)	12 (5.8%)	Categorical FCS model	No
Baseline FT3	Continuous	0	0	—	No
Baseline FT4	Continuous	0	0	—	Yes
Baseline TPOAb	Continuous	3 (1.4%)	3 (1.4%)	PMM	Yes
Baseline TgAb	Continuous	3 (1.4%)	3 (1.4%)	PMM	No
Baseline TRAb	Continuous	0	0	—	Yes
FT3–3m percentage decrease per adj.cum.MMI	Continuous	0	1 (0.5%)	PMM	Yes
FT4–3m percentage decrease per adj.cum.MMI	Continuous	0	1 (0.5%)	PMM	No
TSH–3m percentage decrease per adj.cum.MMI	Continuous	0	1 (0.5%)	PMM	No
TPOAb–3m percentage decrease per adj.cum.MMI	Continuous	8 (3.7%)	14 (6.7%)	PMM	Yes
TgAb–3m percentage decrease per adj.cum.MMI	Continuous	8 (3.7%)	14 (6.7%)	PMM	No
TRAb–3m percentage decrease per adj.cum.MMI	Continuous	6 (2.8%)	9 (4.3%)	PMM	Yes

Multiple imputation by chained equations was performed separately in the development and validation cohorts under fully conditional specification. Predictive mean matching (PMM) was used for continuous variables. Categorical fully conditional specification (FCS) models incorporated interactions among categorical predictors. FT3, free triiodothyronine; FT4, free thyroxine; GD, Graves' disease; GO, Graves' orbitopathy; MI, multiple imputation; MMI, Methimazole; TgAb, thyroglobulin antibody; TPOAb, thyroid peroxidase antibody; TRAb, thyroid stimulating hormone receptor antibody; TSH, thyroid stimulating hormone.

Table S18 Tuning strategy and selected hyperparameters for candidate models

Algorithm	Feature set	Tuning grid	Selected hyperparameters
Clinical-only model			
Logistic	Clinical features	No hyperparameter grid; standard logistic regression	No penalty; no tuned hyperparameter
Ridge	Clinical features	*alpha* fixed at 0; *lambda* grid, *n*=18 (1.00e-04 to 50.0, log-spaced)	*alpha*=0; *lambda*=0.1040
Random forest	Clinical features	*Mtry* grid: 1, 2, 3; *ntree* fixed at 500	*Mtry*=1; *ntree*=500; *nodesize*=1 default; *maxnodes* not limited
XGBoost	Clinical features	Restricted grid, 12 candidates; *nrounds*: 50, 75, 100, 150; *max_depth*: 2; *eta*: 0.01, 0.03, 0.05; *gamma*: 0.5, 1; *subsample*: 0.75, 0.8; *colsample_bytree*: 0.75, 0.8; *min_child_weight*: 1, 3	*Nrounds*=50; *max_depth*=2; *eta*=0.05; *subsample*=0.75; *colsample_bytree*=0.75; *min_child_weight*=1; *gamma*=1.00
SVM	Clinical features	Radial kernel grid; *sigma*: 0.005, 0.01, 0.03; *C*: 0.1, 0.25, 0.5, 1	Radial kernel; *C*=0.50; *sigma*=0.010
Integrated model			
Random forest	Clinical features + candidate SNP risk score	*Mtry* grid: 1, 2, 3; *ntree* fixed at 500	*Mtry*=1; *ntree*=500; *nodesize*=1 default; *maxnodes* not limited

All models were tuned using the train function from the caret package with 10-fold cross-validation; the selection metric was negative log loss. SNP, single nucleotide polymorphism; SVM, support vector machine; XGBoost, extreme gradient boosting.
