# Supplementary material for: Genetic polymorphisms and early responses predict refractory hyperthyroidism during antithyroid drug therapy
Source: iScience. 2026 Oct 8;29(11):117780. doi: 10.1016/j.isci.2026.117780 (PMC13676917; doi:10.1016/j.isci.2026.117780)
Supplement: Supplementary file 2 [file mmc2.zip › RCT Analysis SuppInfo.pdf]

Blinding

The study adopted an open-label design, meaning that investigators, statisticians, and patients were all aware of group assignments.

Randomization

Randomization sequences were independently generated by professional statisticians who were not involved in data analysis or statistical decision-making.

Sample size calculation

A priori sample size estimation was performed using G*Power version 3.1 software, with a significance level (α) of 0.05 and statistical power of 80%. Based on outcome data from previous studies, difference analysis indicated that 105 participants per group were required to reliably detect a 20% clinical difference. To account for potential attrition, an additional 15% was added to each group. Consequently, 120 participants were included in each group, yielding a total sample size of 240 participants for the development cohort. No predefined sample size was established for the validation cohort.

Study protocol

The development cohort was divided into a conventional therapy group and an intensive therapy group, whereas all participants in the validation cohort received standard clinical treatment regimens. Methimazole (MMI, Merck, Germany) was used as the antithyroid drug for all patients with Graves' disease (GD). Levothyroxine (LT_4) was prescribed to supplement thyroid hormone levels in cases of drug-induced hypothyroidism. The concomitant use of β-blockers, leukocyte-stimulating agents, or platelet-stimulating agents was permitted; however, the use of medications that interfere with MMI efficacy or are contraindicated in GD was strictly prohibited. Detailed treatment regimens are described below.

1. **Conventional therapy group**

MMI therapy was initiated at stratified doses according to baseline serum free thyroxine (FT4) levels: 30 mg/day for participants with FT4 ≥ 3 times the upper limit of normal (ULN; 22 pmol/L) and 15 mg/day for those with FT4 < 3× ULN. Thyroid function and thyroid autoantibody levels were

evaluated every 4 weeks. Dose reduction was initiated when TSH exceeded the ULN (4.2 μIU/mL) or when FT4 dropped below the lower limit of normal (LLN; 12.0 pmol/L). The tapering schedules were as follows: 30→20→10→5→2.5 mg/day for participants initially receiving 30 mg/day, and 15→10→5→2.5 mg/day for those starting at 15 mg/day. If a marked elevation in thyroid-stimulating hormone (TSH, e.g., > 100 μIU/mL) occurred during treatment, the tapering rate was accelerated, and skipping one to two dose levels was permitted.

Drug withdrawal was considered after 12–18 months of conventional therapy if thyroid function (free triiodothyronine [FT3], FT4, and TSH) remained within normal ranges or exhibited mild drug-induced hypothyroidism, and if thyroid-stimulating hormone receptor antibody (TRAb) was negative. For participants with persistently positive TRAb (> 1.5 IU/L) beyond 2 years of therapy, the decision to extend treatment or attempt drug withdrawal was made at the discretion of the treating physician, taking into account the patient's clinical status and willingness to continue therapy.

2. Intensive therapy group

The intensive regimen was a prospectively specified, exploratory modified partial block-and-replace strategy rather than a standard antithyroid drug (ATD) treatment approach. It was based on the premise that thyroid peroxidase antibody (TPOAb), thyroglobulin antibody (TgAb), and TRAb may provide complementary information on the underlying thyroid autoimmune status in GD. Therefore, in addition to TRAb, TPOAb and TgAb were incorporated as antibody targets in the intensive regimen. Normalization of all three antibodies was used as a protocol-defined immunological milestone for transition from maintenance treatment to final tapering and withdrawal. The purpose was to test whether pursuing more comprehensive serological remission across thyroid autoantibodies, rather than relying on thyroid-function control and TRAb alone, could improve long-term outcomes. This antibody-targeted component was specific to the intensive regimen and did not apply to conventional dose titration.

The initial MMI dosage was identical to that used in the conventional therapy group, and thyroid function and thyroid autoantibody levels were monitored every 4 weeks. Dose reduction was initiated when serum TSH exceeded the ULN or when FT4 fell below the LLN. For participants initially receiving 30 mg/day, the MMI dose was tapered to 15 mg/day following a 30→20→15 mg/day sequence and then maintained at 15 mg/day, whereas participants who started at 15 mg/day continued on the same dose. LT_4 (Merck, Germany) was added to correct hypothyroidism that developed during treatment. Once TPOAb, TgAb, and TRAb levels all normalized, the existing regimen was maintained for 6 months, after

which MMI was gradually reduced by approximately one-third per month until complete withdrawal.

Special circumstances encountered during intensive therapy were managed according to predefined adjustment protocols. If hypothyroidism did not develop while maintaining 15 mg/day of MMI, the dose was increased to 20 mg/day to ensure adequate thyroid function control. If all three thyroid antibodies failed to normalize within 6 months of maintenance or reverted to positivity after initial normalization, the MMI dose was increased to 20 mg/day; once all antibody levels normalized again, this dosage was maintained for an additional 6 months before resuming the standard tapering schedule. For participants with persistently positive thyroid antibodies across all three markers after more than 2 years of treatment, the decision to extend therapy or initiate gradual dose reduction and withdrawal was made at the discretion of the treating physician, based on clinical judgment and the patient's willingness to continue therapy.

Follow-up

Follow-up visits were performed every 4–8 weeks during treatment. At each visit, thyroid function, thyroid autoantibody levels, complete blood count, and liver function were evaluated. Adverse events were recorded in detail using a standardized case report form. After drug withdrawal, follow-up assessments were conducted every 3–6 months for at least 1 year, focusing on thyroid function and autoantibody monitoring to dynamically evaluate disease recurrence.

Outcomes of the randomized controlled trial (RCT) cohort

Results from the intention-to-treat (ITT) population indicated no significant differences between the intensive therapy group and the conventional therapy group in either the primary outcome (refractory rate, relative risk [RR] = 0.98, $P > 0.05$) or the secondary outcomes (post-withdrawal recurrence rate, RR = 0.97, $P > 0.05$; persistent medication rate—defined as the proportion of patients who did not meet withdrawal criteria after more than 2 years of therapy—RR = 1.38, $P > 0.05$) (Table S3).

Outcomes in the per-protocol (PP) population were consistent with those in the ITT population. No significant differences were observed in the refractory rate, post-withdrawal recurrence rate, or persistent medication rate between the two groups ($P > 0.05$) (Table S4). These findings confirm that intensive and conventional therapy produced no significant differences in the outcomes of refractory GD.

Exploratory thyroid-autoantibody outcomes in the intensive-therapy group

The intensive regimen prospectively tested the hypothesis that sustained moderate-dose MMI treatment using a modified partial block-and-replace strategy, with LT4 supplementation when necessary, might exert immunomodulatory effects, promote broader normalization of thyroid autoimmunity, and reduce relapse. After biochemical control was achieved, further MMI tapering was withheld until TPOAb, TgAb, and TRAb had all become negative. Simultaneous negativity of all three antibodies was therefore a protocol-defined requirement for transition from maintenance treatment to final tapering.

In the per-protocol intensive-therapy population ($N = 109$), all participants had positive TRAb (≥1.5 IU/L) at baseline, whereas 32 participants (29.4%) already had TPOAb levels within the local reference range (<34.0 IU/mL) and 47 (43.1%) already had TgAb levels within the local reference range (<115.0 IU/mL). Thus, TPOAb or TgAb negativity during follow-up did not necessarily represent treatment-induced seroconversion.

Within 24 months, 84 of 109 participants (77.1%) achieved TRAb negativity. Among participants who were antibody-positive at baseline, 30 of 77 (39.0%) achieved TPOAb seroconversion and 11 of 62 (17.7%) achieved TgAb seroconversion. Overall, 62 participants (56.9%) achieved or maintained TPOAb negativity, 58 (53.2%) achieved or maintained TgAb negativity, and 46 (42.2%) achieved simultaneous negativity of all three antibodies. Among the 84 participants who achieved TRAb negativity, 38 (45.2%) still had positive TPOAb and/or TgAb, including 14 (16.7%) with isolated TgAb positivity.

Participants whose TRAb became negative while TPOAb and/or TgAb remained positive continued moderate-dose MMI treatment without further protocol-directed tapering. When antibody positivity persisted for more than 2 years, continued treatment or withdrawal was individualized through discussion between the physician and the patient. Participants willing to continue remained on treatment, whereas those unwilling to continue discontinued MMI despite not meeting the protocol-defined antibody milestone.

Despite the observed antibody responses, the intensive regimen did not improve the primary or secondary clinical outcomes compared with conventional treatment. These findings did not support the original hypothesis that pursuing broader thyroid-autoantibody normalization through this regimen would reduce refractory disease or relapse. Therefore, TPOAb and TgAb negativity should not be interpreted as established targets for routine ATD tapering or treatment withdrawal.

Adverse events of the RCT cohort

Safety analysis showed no statistically significant difference in the incidence of any type of adverse event between the two groups ($P > 0.05$). Elevated liver enzymes (55.8% vs. 51.7%), neutropenia (22.5% vs. 25.0%), and skin rash (13.3% vs. 11.7%) were the predominant adverse events. Other adverse events, including elevated bilirubin and thrombocytopenia, occurred infrequently and were similarly distributed between groups, indicating that the two regimens were comparable in safety (Table S5).
