# Supplementary material for: Genetic polymorphisms and early responses predict refractory hyperthyroidism during antithyroid drug therapy
Source: iScience. 2026 Oct 8;29(11):117780. doi: 10.1016/j.isci.2026.117780 (PMC13676917; doi:10.1016/j.isci.2026.117780)
Supplement: Supplementary file 3 [file mmc3.zip › R Code SuppInfo.pdf]

Supplemental R Code

Scope

This supplement provides the executable R scripts for clinical feature selection, model tuning, final random-forest fitting, bootstrap resampling, temporal validation, risk reclassification, and the CD40 sensitivity analysis. The scripts do not contain participant-level data, fitted model objects, prediction files, or generated result tables.

Software and Inputs

The scripts were prepared for R 4.4 or later. Required packages are pROC, randomForest, caret, glmnet, xgboost, kernlab, and mice. The downstream scripts expect completed and categorised development- and temporal-validation data files named development_analysis_ready.csv and validation_analysis_ready.csv in a data subdirectory.

Analytic Workflow

1. Specify the multiple-imputation procedure (Code S1).
2. Perform univariable screening, VIF assessment, and LASSO with 10-fold cross-validation (Code S2).
3. Tune five candidate clinical algorithms using 10-fold cross-validation and log-loss selection (Code S3).
4. Fit the selected clinical-only and integrated random-forest models (Code S4).
5. Summarise fixed-final-model bootstrap AUCs and evaluate temporal validation performance (Code S5).
6. Evaluate risk reclassification and the CD40-only sensitivity analysis (Codes S6-S7).

Code Files

File	Purpose
Code S0. Shared helper functions (Code_S0_helper_functions.R)	Shared input preparation and performance functions.
Code S1. Multiple-imputation specification (Code_S1_MICE_specification.R)	Cohort-specific MICE specification; SNP variables are not imputed.
Code S2. Clinical feature selection (Code_S2_clinical_feature_selection.R)	Univariable screening, VIF assessment, and LASSO selection.
Code S3. Candidate-model tuning (Code_S3_candidate_model_tuning.R)	10-fold cross-validation tuning of five candidate algorithms.
Code S4. Final random-forest model fitting (Code_S4_final_RF_model_fitting.R)	Final clinical-only and integrated random-forest fitting.
Code S5. Bootstrap resampling and temporal validation (Code_S5_bootstrap_and_temporal_validation.R)	Bootstrap AUC summary and temporal validation.
Code S6. Risk reclassification analysis (Code_S6_reclassification_analysis.R)	Risk-stratum directional reclassification analysis.
Code S7. CD40 sensitivity analysis (Code_S7_CD40_sensitivity_analysis.R)	CD40-only compared with candidate four-SNP score models.
Code S8. R session information	Exports R session information.

(Code_S8_session_information.R)	
Code S9. Master execution script (Code_S9_run_all.R)	Runs the downstream scripts in sequence.

Reporting note. Development-cohort model performance is apparent performance. The bootstrap analysis resamples predictions from fixed final models and does not repeat feature selection, tuning, or model fitting; it is therefore not a full optimism-corrected internal validation.

Code S0. Shared helper functions

File: Code_S0_helper_functions.R

```r
required_packages <- c("pROC", "randomForest", "caret", "glmnet")

load_required_packages <- function() {
  missing <- required_packages[!vapply(required_packages, requireNamespace, logical(1), quietly = TRUE)]
  if (length(missing) > 0) {
    stop(
      "Install the required R packages before running the analysis: ",
      paste(missing, collapse = ", "),
      call. = FALSE
    )
  }
}

package_root <- function() {
  root <- getwd()
  if (!file.exists(file.path(root, "data", "development_analysis_ready.csv"))) {
    stop("Run Code_S9_run_all.R from the code-supplement root directory.", call. = FALSE)
  }
  root
}

selected_clinical_variables <- c(
  "Age",
  "Initial.Volume",
  "Initial.FT4",
  "Initial.TPOAb",
  "Initial.TRAb",
  "FT3.percentage.decrease.per.mmi",
  "TPOAb.percentage.decrease.per.mmi",
  "TRAb.decrease.percentage.per.mmi"
)

candidate_clinical_variables <- c(
  "Age",
  "Female",
  "Initial.Volume",
  "Initial.FT3",
  "Initial.FT4",
  "Initial.TPOAb",
  "Initial.TgAb",
  "Initial.TRAb",
  "Smoking.History",
```

```
  "Family.History",
  "GO",
  "FT3.percentage.decrease.per.mmi",
  "FT4.percentage.decrease.per.mmi",
  "TSH.percentage.increase.per.mmi",
  "TPOAb.percentage.decrease.per.mmi",
  "TgAb.percentage.decrease.per.mmi",
  "TRAb.decrease.percentage.per.mmi"
)

as_binary <- function(values) {
  values <- suppressWarnings(as.integer(as.character(values)))
  if (!all(values %in% c(0L, 1L))) {
    stop("A binary variable contains values other than 0 and 1.", call. = FALSE)
  }
  values
}

as_cd40_dosage <- function(values) {
  dosage <- suppressWarnings(as.integer(as.character(values)))
  if (!all(dosage %in% 0:2)) stop("CD40 must be coded as 0, 1, or 2.", call. = FALSE)
  dosage
}

prepare_analysis_data <- function(path, require_all_candidates = FALSE) {
  raw <- read.csv(path, check.names = FALSE, stringsAsFactors = FALSE, fileEncoding = "UTF-8-BOM")
  required <- c("Record_ID", "Refractory", selected_clinical_variables, "PRS", "CD40")
  if (require_all_candidates) required <- c("Record_ID", "Refractory", candidate_clinical_variables, "PRS", "CD40")
  absent <- setdiff(required, names(raw))
  if (length(absent) > 0) stop("Missing columns: ", paste(absent, collapse = ", "), call. = FALSE)
  if (anyNA(raw[required])) stop("Analysis-ready data contain missing values in required variables.", call. = FALSE)

  raw$Refractory <- factor(ifelse(as_binary(raw$Refractory) == 1L, "yes", "no"), levels = c("no", "yes"))
  for (variable in intersect(candidate_clinical_variables, names(raw))) {
    raw[[variable]] <- factor(as_binary(raw[[variable]]), levels = c(0, 1), labels = c("0", "1"))
  }
  raw$PRS <- factor(as.integer(raw$PRS), levels = 1:5, labels = as.character(1:5))
  raw$CD40_dosage <- as_cd40_dosage(raw$CD40)
  raw$CD40_factor <- factor(raw$CD40_dosage, levels = 0:2, labels = c("0", "1", "2"))
  raw
}

safe_probability <- function(probability) pmin(pmax(as.numeric(probability), 1e-6), 1 - 1e-6)
```

```
auc_with_ci <- function(outcome, probability) {
  roc_object <- pROC::roc(outcome, safe_probability(probability), levels = c("no", "yes"), direction = "<", quiet = TRUE)
  ci <- suppressMessages(pROC::ci.auc(roc_object))
  c(AUC = as.numeric(pROC::auc(roc_object)), Lower = as.numeric(ci[1]), Upper = as.numeric(ci[3]))
}

calibration_statistics <- function(outcome, probability) {
  observed <- ifelse(outcome == "yes", 1, 0)
  logit_probability <- qlogis(safe_probability(probability))
  c(
    Brier = mean((safe_probability(probability) - observed)^2),
    Calibration_intercept = unname(coef(glm(observed ~ 1 + offset(logit_probability), family = binomial()))[1]),
    Calibration_slope = unname(coef(glm(observed ~ logit_probability, family = binomial()))[2])
  )
}

classification_statistics <- function(outcome, probability, threshold = 0.50) {
  observed <- outcome == "yes"
  predicted <- safe_probability(probability) >= threshold
  tp <- sum(predicted & observed)
  fp <- sum(predicted & !observed)
  tn <- sum(!predicted & !observed)
  fn <- sum(!predicted & observed)
  c(
    TP = tp,
    FP = fp,
    TN = tn,
    FN = fn,
    Accuracy = (tp + tn) / length(outcome),
    Sensitivity = ifelse(tp + fn == 0, NA_real_, tp / (tp + fn)),
    Specificity = ifelse(tn + fp == 0, NA_real_, tn / (tn + fp)),
    PPV = ifelse(tp + fp == 0, NA_real_, tp / (tp + fp)),
    NPV = ifelse(tn + fn == 0, NA_real_, tn / (tn + fn)),
    F1 = ifelse(2 * tp + fp + fn == 0, NA_real_, 2 * tp / (2 * tp + fp + fn))
  )
}

performance_row <- function(cohort, model, outcome, probability, threshold = 0.50) {
  auc <- auc_with_ci(outcome, probability)
  calibration <- calibration_statistics(outcome, probability)
  classification <- classification_statistics(outcome, probability, threshold)
  data.frame(
    Cohort = cohort,
    Model = model,
```

```
    N = length(outcome),
    Events = sum(outcome == "yes"),
    AUC = unname(auc["AUC"]),
    AUC_95CI_lower = unname(auc["Lower"]),
    AUC_95CI_upper = unname(auc["Upper"]),
    Brier_score = unname(calibration["Brier"]),
    Calibration_intercept = unname(calibration["Calibration_intercept"]),
    Calibration_slope = unname(calibration["Calibration_slope"]),
    t(classification),
    check.names = FALSE
  )
}

write_csv <- function(data, path) write.csv(data, path, row.names = FALSE, na = "")
```

Code S1. Multiple-imputation specification

File: Code_S1_MICE_specification.R

```r
# This script records the cohort-specific MICE procedure used before model development.
# Run it on the corresponding pre-imputation cohort dataset when re-creating preprocessing.

if (!requireNamespace("mice", quietly = TRUE)) {
  stop("Install the mice package before running this preprocessing script.", call. = FALSE)
}

run_mice <- function(data, continuous_variables, binary_variables, seed = 20260706) {
  variables <- unique(c(continuous_variables, binary_variables, "Refractory"))
  absent <- setdiff(variables, names(data))
  if (length(absent) > 0) stop("Missing variables: ", paste(absent, collapse = ", "), call. = FALSE)
  input <- data[, variables, drop = FALSE]
  input$Refractory <- factor(input$Refractory, levels = c(0, 1))
  for (variable in binary_variables) input[[variable]] <- factor(input[[variable]], levels = c(0, 1))

  methods <- mice::make.method(input)
  methods[continuous_variables] <- "pmm"
  methods[binary_variables] <- "logreg"
  methods["Refractory"] <- ""
  predictors <- mice::quickpred(input, mincor = 0, minpuc = 0)
  predictors["Refractory", ] <- 0

  mice::mice(
    input,
    m = 20,
    maxit = 50,
    method = methods,
    predictorMatrix = predictors,
    seed = seed,
    printFlag = FALSE
  )
}

# The packaged CSV files are the completed, categorised datasets used for model fitting.
# This function intentionally does not impute SNP variables: they are retained as observed genotypes.
```

Code S2. Clinical feature selection

File: Code_S2_clinical_feature_selection.R

```r
source("Code_S0_helper_functions.R")
load_required_packages()
root <- package_root()
set.seed(123)

development <- prepare_analysis_data(file.path(root, "data", "development_analysis_ready.csv"), require_all_candidates = TRUE)
outcome <- ifelse(development$Refractory == "yes", 1, 0)

univariable <- do.call(rbind, lapply(candidate_clinical_variables, function(variable) {
  fit <- glm(reformulate(variable, response = "outcome"), data = development, family = binomial())
  coefficient <- summary(fit)$coefficients[2, ]
  data.frame(
    Variable = variable,
    Odds_ratio = exp(coefficient["Estimate"]),
    P_value = coefficient["Pr(>|z|)"],
    stringsAsFactors = FALSE
  )
}))
write_csv(univariable, file.path(root, "outputs", "univariable_screening.csv"))

screened_variables <- univariable$Variable[univariable$P_value < 0.10]
if (length(screened_variables) == 0) stop("No variables met the prespecified P < 0.10 screen.", call. = FALSE)

predictor_matrix <- do.call(cbind, lapply(screened_variables, function(variable) as.integer(as.character(development[[variable]]))))
colnames(predictor_matrix) <- screened_variables
lasso_cv <- glmnet::cv.glmnet(scale(predictor_matrix), outcome, family = "binomial", alpha = 1, nfolds = 10)
coefficients <- as.matrix(coef(lasso_cv, s = "lambda.1se"))
selected <- rownames(coefficients)[coefficients[, 1] != 0]
selected <- setdiff(selected, "(Intercept)")

selection <- data.frame(
  Variable = selected,
  Coefficient_at_lambda_1se = coefficients[selected, 1],
  Lambda_1se = lasso_cv$lambda.1se,
  stringsAsFactors = FALSE
)
write_csv(selection, file.path(root, "outputs", "lasso_selection_lambda_1se.csv"))
write_csv(data.frame(Screened_variable = screened_variables), file.path(root, "outputs", "variables_with_univariable_p_lt_0_10.csv"))
cat("Feature screening complete. lambda.1se =", format(lasso_cv$lambda.1se, digits = 5), "\n")
```

Code S3. Candidate-model tuning

File: Code_S3_candidate_model_tuning.R

```
source("Code_S0_helper_functions.R")
load_required_packages()
root <- package_root()
set.seed(20260706)

development <- prepare_analysis_data(file.path(root, "data", "development_analysis_ready.csv"))
model_data <- development[, c("Refractory", selected_clinical_variables)]
control <- caret::trainControl(
  method = "cv", number = 10, classProbs = TRUE,
  summaryFunction = caret::mnLogLoss, savePredictions = "final"
)

xgb_grid <- data.frame(
  nrounds = c(50, 50, 50, 75, 75, 75, 100, 100, 100, 150, 150, 150),
  max_depth = 2, eta = rep(c(0.01, 0.03, 0.05), 4),
  gamma = rep(c(0.5, 1), each = 3, length.out = 12),
  colsample_bytree = rep(c(0.75, 0.80), each = 6),
  min_child_weight = rep(c(1, 3), times = 6),
  subsample = rep(c(0.75, 0.80), times = 6)
)

fits <- list(
  Logistic = caret::train(Refractory ~ ., data = model_data, method = "glm", metric = "logLoss", trControl = control),
  Ridge = caret::train(Refractory ~ ., data = model_data, method = "glmnet", metric = "logLoss", trControl = control,
    preProcess = c("center", "scale"), tuneGrid = expand.grid(alpha = 0, lambda = exp(seq(log(1e-4), log(50), length.out = 18)))),
  Random_forest = caret::train(Refractory ~ ., data = model_data, method = "rf", metric = "logLoss", trControl = control,
    tuneGrid = data.frame(mtry = 1:3), ntree = 500, importance = TRUE),
  XGBoost = caret::train(Refractory ~ ., data = model_data, method = "xgbTree", metric = "logLoss", trControl = control,
    tuneGrid = xgb_grid, verbosity = 0),
  SVM = caret::train(Refractory ~ ., data = model_data, method = "svmRadial", metric = "logLoss", trControl = control,
    preProcess = c("center", "scale"), tuneGrid = expand.grid(sigma = c(0.005, 0.01, 0.03), C = c(0.1, 0.25, 0.5, 1)))
)

best <- do.call(rbind, lapply(names(fits), function(name) {
  fit <- fits[[name]]
  result <- fit$results[which.min(fit$results$logLoss), , drop = FALSE]
  cbind(Algorithm = name, result)
}))
write_csv(best, file.path(root, "outputs", "candidate_model_tuning_results.csv"))
saveRDS(fits, file.path(root, "outputs", "candidate_model_caret_fits.rds"))
cat("Candidate-model tuning complete.\n")
```

Code S4. Final random-forest model fitting

File: Code_S4_final_RF_model_fitting.R

```r
source("Code_S0_helper_functions.R")
load_required_packages()
root <- package_root()
set.seed(20260706)

development <- prepare_analysis_data(file.path(root, "data", "development_analysis_ready.csv"))
clinical_data <- development[, c("Refractory", selected_clinical_variables)]
integrated_data <- development[, c("Refractory", selected_clinical_variables, "PRS")]

clinical_rf <- randomForest::randomForest(Refractory ~ ., data = clinical_data, mtry = 1, ntree = 500, importance = TRUE)
set.seed(20260706)
integrated_rf <- randomForest::randomForest(Refractory ~ ., data = integrated_data, mtry = 1, ntree = 500, importance = TRUE)

clinical_probability <- predict(clinical_rf, clinical_data[, selected_clinical_variables], type = "prob")[, "yes"]
integrated_probability <- predict(integrated_rf, integrated_data[, c(selected_clinical_variables, "PRS")], type = "prob")[, "yes"]

saveRDS(clinical_rf, file.path(root, "model", "clinical_rf.rds"))
saveRDS(integrated_rf, file.path(root, "model", "clinical_candidate_snp_score_rf.rds"))
write_csv(
  rbind(
    performance_row("Development apparent", "Clinical RF", development$Refractory, clinical_probability),
    performance_row("Development apparent", "Clinical + candidate SNP risk score RF", development$Refractory, integrated_probability)
  ),
  file.path(root, "outputs", "development_final_rf_performance.csv")
)
write_csv(data.frame(
  Record_ID = development$Record_ID,
  Refractory = development$Refractory,
  Clinical_RF_probability = clinical_probability,
  Clinical_candidate_SNP_score_RF_probability = integrated_probability
), file.path(root, "outputs", "development_final_rf_predictions.csv"))
cat("Final random-forest models fitted and saved.\n")
```

Code S5. Bootstrap resampling and temporal validation

File: Code_S5_bootstrap_and_temporal_validation.R

```
source("Code_S0_helper_functions.R")
load_required_packages()
root <- package_root()
set.seed(20260706)

clinical_rf <- readRDS(file.path(root, "model", "clinical_rf.rds"))
integrated_rf <- readRDS(file.path(root, "model", "clinical_candidate_snp_score_rf.rds"))
development <- prepare_analysis_data(file.path(root, "data", "development_analysis_ready.csv"))
validation <- prepare_analysis_data(file.path(root, "data", "validation_analysis_ready.csv"))

predict_models <- function(data) {
  clinical_probability <- predict(clinical_rf, data[, selected_clinical_variables], type = "prob")[, "yes"]
  integrated_probability <- predict(integrated_rf, data[, c(selected_clinical_variables, "PRS")], type = "prob")[, "yes"]
  data.frame(Clinical_RF_probability = clinical_probability, Clinical_candidate_SNP_score_RF_probability = integrated_probability)
}

development_probabilities <- predict_models(development)
validation_probabilities <- predict_models(validation)

bootstrap_auc <- function(outcome, probability, n_resamples = 1000, seed = 20260706) {
  set.seed(seed)
  replicate(n_resamples, {
    index <- sample.int(length(outcome), replace = TRUE)
    if (length(unique(outcome[index])) < 2) return(NA_real_)
    unname(auc_with_ci(outcome[index], probability[index])["AUC"])
  })
}

clinical_bootstrap <- bootstrap_auc(development$Refractory, development_probabilities$Clinical_RF_probability)
integrated_bootstrap <- bootstrap_auc(development$Refractory, development_probabilities$Clinical_candidate_SNP_score_RF_probability)
bootstrap_summary <- data.frame(
  Model = c("Clinical RF", "Clinical + candidate SNP risk score RF"),
  Mean_AUC = c(mean(clinical_bootstrap, na.rm = TRUE), mean(integrated_bootstrap, na.rm = TRUE)),
  Median_AUC = c(median(clinical_bootstrap, na.rm = TRUE), median(integrated_bootstrap, na.rm = TRUE)),
  AUC_2_5th_percentile = c(quantile(clinical_bootstrap, 0.025, na.rm = TRUE), quantile(integrated_bootstrap, 0.025, na.rm = TRUE)),
  AUC_97_5th_percentile = c(quantile(clinical_bootstrap, 0.975, na.rm = TRUE), quantile(integrated_bootstrap, 0.975, na.rm = TRUE))
)
write_csv(bootstrap_summary, file.path(root, "outputs", "bootstrap_auc_summary.csv"))

validation_performance <- rbind(
  performance_row("Temporal validation", "Clinical RF", validation$Refractory, validation_probabilities$Clinical_RF_probability),
  performance_row("Temporal validation", "Clinical + candidate SNP risk score RF", validation$Refractory,
```

```
validation_probabilities$Clinical_candidate_SNP_score_RF_probability)
)
write_csv(validation_performance, file.path(root, "outputs", "temporal_validation_final_rf_performance.csv"))
write_csv(data.frame(
  Record_ID = validation$Record_ID,
  Refractory = validation$Refractory,
  validation_probabilities
), file.path(root, "outputs", "temporal_validation_final_rf_predictions.csv"))
cat("Bootstrap resampling and temporal validation complete.\n")
```

Code S6. Risk reclassification analysis

File: Code_S6_reclassification_analysis.R

```
source("Code_S0_helper_functions.R")
load_required_packages()
root <- package_root()

risk_group <- function(probability) cut(
  probability,
  breaks = c(-Inf, 0.30, 0.60, Inf),
  labels = c("Low (<0.30)", "Intermediate (0.30-0.59)", "High (>=0.60)"),
  right = FALSE
)

reclassification_summary <- function(path, cohort) {
  data <- read.csv(path, check.names = FALSE, stringsAsFactors = FALSE, fileEncoding = "UTF-8-BOM")
  old_group <- risk_group(data$Clinical_RF_probability)
  new_group <- risk_group(data$Clinical_candidate_SNP_score_RF_probability)
  direction <- as.integer(new_group) - as.integer(old_group)
  event <- data$Refractory == "yes"
  correct <- (event & direction > 0) | (!event & direction < 0)
  incorrect <- (event & direction < 0) | (!event & direction > 0)
  do.call(rbind, lapply(levels(old_group), function(group) {
    index <- old_group == group
    data.frame(
      Cohort = cohort,
      Clinical_only_risk_stratum = group,
      N = sum(index),
      Events = sum(event[index]),
      Reclassified = sum(direction[index] != 0),
      Correct_direction = sum(correct[index]),
      Incorrect_direction = sum(incorrect[index]),
      Net_correct_contribution = (sum(correct[index]) - sum(incorrect[index])) / sum(index),
      check.names = FALSE
    )
  }))
}

summary <- rbind(
  reclassification_summary(file.path(root, "outputs", "development_final_rf_predictions.csv"), "Development"),
  reclassification_summary(file.path(root, "outputs", "temporal_validation_final_rf_predictions.csv"), "Temporal validation")
)
write_csv(summary, file.path(root, "outputs", "reclassification_directional_correctness.csv"))
cat("Risk reclassification analysis complete.\n")
```

Code S7. CD40 sensitivity analysis

File: Code_S7_CD40_sensitivity_analysis.R

```
source("Code_S0_helper_functions.R")
load_required_packages()
root <- package_root()
set.seed(20260706)

development <- prepare_analysis_data(file.path(root, "data", "development_analysis_ready.csv"))
validation <- prepare_analysis_data(file.path(root, "data", "validation_analysis_ready.csv"))

fit_models <- function(data) {
  clinical <- randomForest::randomForest(
    Refractory ~ ., data = data[, c("Refractory", selected_clinical_variables)],
    mtry = 1, ntree = 500, importance = TRUE
  )
  set.seed(20260706)
  cd40 <- randomForest::randomForest(
    Refractory ~ ., data = data[, c("Refractory", selected_clinical_variables, "CD40_factor")],
    mtry = 1, ntree = 500, importance = TRUE
  )
  set.seed(20260706)
  four_snp <- randomForest::randomForest(
    Refractory ~ ., data = data[, c("Refractory", selected_clinical_variables, "PRS")],
    mtry = 1, ntree = 500, importance = TRUE
  )
  list(clinical = clinical, cd40 = cd40, four_snp = four_snp)
}

predict_models <- function(models, data) {
  data.frame(
    Clinical_RF_probability = predict(models$clinical, data[, selected_clinical_variables], type = "prob")[, "yes"],
    Clinical_CD40_RF_probability = predict(models$cd40, data[, c(selected_clinical_variables, "CD40_factor")], type = "prob")[, "yes"],
    Clinical_candidate_SNP_score_RF_probability = predict(models$four_snp, data[, c(selected_clinical_variables, "PRS")], type = "prob")[,
"yes"]
  )
}

models <- fit_models(development)
development_probability <- predict_models(models, development)
validation_probability <- predict_models(models, validation)

comparison_rows <- function(cohort, outcome, probability) {
  rbind(
    performance_row(cohort, "Clinical-only RF", outcome, probability$Clinical_RF_probability),
```

```
    performance_row(cohort, "Integrated RF with CD40", outcome, probability$Clinical_CD40_RF_probability),
    performance_row(cohort, "Integrated RF with candidate four-SNP risk score", outcome,
probability$Clinical_candidate_SNP_score_RF_probability)
  )
}

performance <- rbind(
  comparison_rows("Development apparent", development$Refractory, development_probability),
  comparison_rows("Temporal validation", validation$Refractory, validation_probability)
)

delong <- function(outcome, cd40_probability, four_snp_probability) {
  cd40_roc <- pROC::roc(outcome, cd40_probability, levels = c("no", "yes"), direction = "<", quiet = TRUE)
  four_snp_roc <- pROC::roc(outcome, four_snp_probability, levels = c("no", "yes"), direction = "<", quiet = TRUE)
  test <- pROC::roc.test(four_snp_roc, cd40_roc, method = "delong", paired = TRUE)
  data.frame(
    Delta_AUC_four_SNP_minus_CD40 = as.numeric(pROC::auc(four_snp_roc) - pROC::auc(cd40_roc)),
    DeLong_P = as.numeric(test$p.value)
  )
}

comparison <- rbind(
  cbind(Cohort = "Development apparent", delong(development$Refractory, development_probability$Clinical_CD40_RF_probability,
development_probability$Clinical_candidate_SNP_score_RF_probability)),
  cbind(Cohort = "Temporal validation", delong(validation$Refractory, validation_probability$Clinical_CD40_RF_probability,
validation_probability$Clinical_candidate_SNP_score_RF_probability))
)

dir.create(file.path(root, "outputs"), recursive = TRUE, showWarnings = FALSE)
write_csv(performance, file.path(root, "outputs", "CD40_vs_candidate_four_SNP_RF_performance.csv"))
write_csv(comparison, file.path(root, "outputs", "CD40_vs_candidate_four_SNP_RF_comparison.csv"))
cat("CD40 sensitivity analysis complete.\n")
```

Code S8. R session information

File: Code_S8_session_information.R

```
root <- getwd()
capture.output(sessionInfo(), file = file.path(root, "outputs", "R_session_info.txt"))
cat("R session information saved.\n")
```

Code S9. Master execution script

File: Code_S9_run_all.R

```
if (!file.exists("Code_S0_helper_functions.R")) stop("Run this script from the code-supplement root directory.", call. = FALSE)
source("Code_S2_clinical_feature_selection.R")
source("Code_S3_candidate_model_tuning.R")
source("Code_S4_final_RF_model_fitting.R")
source("Code_S5_bootstrap_and_temporal_validation.R")
source("Code_S6_reclassification_analysis.R")
source("Code_S7_CD40_sensitivity_analysis.R")
source("Code_S8_session_information.R")
cat("All code-supplement analyses completed.\n")
```
